# Object Detection, Recognition, and Tracking Algorithms for ADASs—A Study on Recent Trends

**DOI:** 10.3390/s24010249

**Published:** 2023-12-31

**Authors:** Vinay Malligere Shivanna, Jiun-In Guo

**Affiliations:** 1 Department of Electrical Engineering, Institute of Electronics, National Yang-Ming Chiao Tung University, Hsinchu City 30010, Taiwan; jiguo@nycu.edu.tw; 2 Pervasive Artificial Intelligence Research (PAIR) Labs, National Yang Ming Chiao Tung University, Hsinchu City 30010, Taiwan; 3 eNeural Technologies Inc., Hsinchu City 30010, Taiwan

**Keywords:** object detection, object tracking, advanced driver assistance system (ADAS), deep learning

## Abstract

Advanced driver assistance systems (ADASs) are becoming increasingly common in modern-day vehicles, as they not only improve safety and reduce accidents but also aid in smoother and easier driving. ADASs rely on a variety of sensors such as cameras, radars, lidars, and a combination of sensors, to perceive their surroundings and identify and track objects on the road. The key components of ADASs are object detection, recognition, and tracking algorithms that allow vehicles to identify and track other objects on the road, such as other vehicles, pedestrians, cyclists, obstacles, traffic signs, traffic lights, etc. This information is then used to warn the driver of potential hazards or used by the ADAS itself to take corrective actions to avoid an accident. This paper provides a review of prominent state-of-the-art object detection, recognition, and tracking algorithms used in different functionalities of ADASs. The paper begins by introducing the history and fundamentals of ADASs followed by reviewing recent trends in various ADAS algorithms and their functionalities, along with the datasets employed. The paper concludes by discussing the future of object detection, recognition, and tracking algorithms for ADASs. The paper also discusses the need for more research on object detection, recognition, and tracking in challenging environments, such as those with low visibility or high traffic density.

## 1. Introduction

Advanced driver assistance systems (ADASs) are a group of electronic technologies that assist drivers in driving and parking functions. Through a safe human–machine interface, ADASs increase car and road safety. They use automated technology, such as sensors and cameras, to detect nearby obstacles or driver errors, and respond or issue alerts accordingly. They can enable various levels of autonomous driving, depending on the features installed in the car.

ADASs use a variety of sensors such as cameras, radar, lidar, and a combination of these, to detect objects and conditions around the vehicle. The sensors send data to a computing system, which then analyzes the data and determines the best course of action based on the algorithmic design. For instance, if a camera detects a pedestrian in the vehicle’s path, the computing system may trigger the ADAS to sound an alarm or apply the brakes.

The chronicles of ADAS date back to the 1970s [1,2] with the development of the first anti-lock braking system (ABS). Following a slow and steady evolution, additional features such as the lane departure warning system (LDWS) and electronic stability control (ESC) emerged in the 1990s. In recent years, there has been a rapid development of numerous ADASs, with new functionalities being introduced every other day and becoming increasingly prevalent in modern vehicles, as they offer a variety of safety features that aid in preventing accidents, relying on the aforementioned variety of sensors that have made the ADAS a potential system with which to significantly reduce the number of traffic accidents and fatalities. A study by the Insurance Institute for Highway Safety [3] found that different uses of ADASs can reduce the risk of a fatal crash by up to 20–25%. Therefore, ADASs are becoming increasingly common in cars. In 2021, 33% of new cars sold in the United States had ADAS features. This number is expected to grow to 50% by 2030, as ADASs are expected to play a major role in the future of transportation [4]. By helping to prevent accidents and collisions, reducing drivers’ fatigue and stress [5,6], improving fuel efficiency [7,8], making parking easier and more convenient [9] and thereby providing peace of mind to drivers and passengers [5,6], ADASs can save lives and make our roads safer.

**Figure 1:**
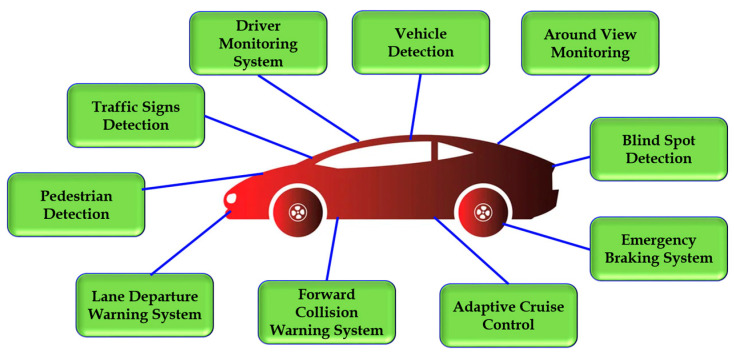
Different features of ADASs.

Additionally, various features of ADASs, as shown in Figure 1, are a crucial part of the development of autonomous driving; in other words, self-driving cars, as autonomous vehicles, rely on the performance and efficiency of ADASs to detect objects and conditions in their surroundings in real-world scenarios. Self-driving cars use a combination of ADASs and artificial intelligence to drive themselves. Therefore, ADASs are continuing to play an important role in the development of autonomous driving as the technology matures.

The basic functionalities of ADASs are object detection, recognition, and tracking. Numerous algorithms allow vehicles to detect and recognize—in other words, to identify and then track—other objects on the road, such as vehicles, pedestrians, cyclists, traffic signs, lanes, probable obstacles on the road, and more; warn the driver of potential hazards; and/or take evasive action automatically.

There are a number of different object detection, recognition, and tracking algorithms that have been developed for ADASs. These algorithms can be broadly classified into two main categories: traditional methods and deep learning (DL) methods, as discussed in detail in Section 1.3.

This paper attempts to provide a comprehensive review of recent trends in different algorithms for various ADAS functions. The paper begins by discussing the challenges of object detection, recognition, and tracking in ADAS applications. The paper then discusses the different types of sensors used in ADASs and different types of object detection, recognition, and tracking algorithms that have been developed for various ADAS methodologies and datasets used to train and test the methods. The paper concludes by discussing the future trends in object detection, recognition, and tracking for ADASs.

### 1.1. Basic Terminologies

Before diving into the main objective of the paper, the section below introduces some of the basic terminologies commonly used in the field of ADAS research:

- Image processing is the process of manipulating digital images to improve their quality or extract useful information from them. Image processing techniques are commonly used in ADASs for object detection, recognition, and tracking tasks;
- Object detection is the task of identifying and locating objects in a scene, such as vehicles, pedestrians, traffic signs, and other objects that could pose a hazard to the driver;
- Object tracking involves following the movement of vehicles, pedestrians, and other objects over time to predict their future trajectories;
- Image segmentation is the task of dividing an image into different regions, each of which corresponds to a different object or part of an object such as the bumper, hood, and wheels and other objects such as pedestrians, traffic signs, lanes, forward objects, and so on;
- Feature extraction is the extraction of features like shape, size, color, and so on from an image or a video; these features are used to identify objects or track their movements.
- Classification is the task of assigning a label such as vehicles, pedestrians, traffic signs, or others to an object or several images to categorize the objects;
- Recognition is the task of identifying an object or a region in an image by its name or other attributes.

### 1.2. An Overview of ADASs

The history of ADAS technology can be traced back to the 1970s with the adoption of the anti-lock braking system [10,11]. Early ADASs including electronic stability control, anti-lock brakes, blind spot information systems, lane departure warning, adaptive cruise control, and traction control emerged in the 1900s and 2000s [12,13]. These systems can be affected by mechanical alignment adjustments or damage from a collision requiring automatic reset for these systems after a mechanical alignment is performed.

#### 1.2.1. The Scope of ADASs

ADASs perform a variety of tasks using object detection, recognition, and tracking algorithms which are deemed as falling within the scope of ADASs; namely, (i) vehicle detection, (ii) pedestrian detection, (iii) traffic signs detection (TSD), (iv) driver monitoring system (DMS), (v) lane departure warning system (LDWS), (vi) forward collision warning system (FCWS), (vii) blind-spot detection (BSD), (viii) emergency braking system (EBS), (ix) adaptive cruise control (ACC), and (x) around view monitoring (AVM).

These are some of the most important of the many ADAS features that rely on detection, recognition, and tracking algorithms. These algorithms are constantly being improved as the demand for safer vehicles continues to grow.

#### 1.2.2. The Objectives of Object Detection, Recognition, and Tracking in ADASs

An ADAS system has various functions with different objectives that can be listed as:

- Improving road safety: ADASs can aid in improving road safety by reducing the number of accidents; this is achieved by warning drivers of potential hazards and by taking corrective actions to avoid collisions. For example, a LDWS can warn the driver if they are about to drift out of their lane, while a forward collision warning system can warn the driver if they are about to collide with another vehicle;
- Reducing driver workload: ADASs can help to reduce driver workload by automating some of their driving tasks. This can help to make driving safer and more enjoyable. For example, ACC can automatically maintain a safe distance between the vehicle and the vehicle in front of it, and lane-keeping assist can automatically keep the vehicle centered in its lane;
- Increasing fuel efficiency: ADASs can help to increase fuel efficiency by reducing the need for the driver to brake and accelerate, which is achieved by maintaining a constant speed and by avoiding sudden speed changes. For example, ACC can automatically adjust the speed of the vehicle to maintain a safe distance from the vehicle in front of it, which can help to reduce fuel consumption;
- Providing information about the road environment: ADASs can provide drivers with more information about the road environment, such as the speed of other vehicles, the distance to the nearest object, traffic signs, and the presence of pedestrians or cyclists. This information can help drivers to make better decisions about how to drive and can help to reduce the risk of accidents;
- Assisting drivers with difficult driving tasks: ADASs can assist drivers with difficult driving tasks, such as parking, merging onto a highway, and driving in bad weather conditions, thereby reducing driver workload and enabling safer driving;
- Ensuring a comfortable and enjoyable driving experience: ADASs can provide a more comfortable and enjoyable driving experience by reducing stress and fatigue that drivers experience which can be achieved by automating some of the tasks involved in driving, such as maintaining a constant speed and avoiding sudden changes in speed.

The ADAS algorithms are designed to achieve these objectives by using sensors, such as cameras, radar, lidar, and now a combination of these, to collect data about the road environment. The data thus obtained are processed by the algorithms as per their design to identify and track objects, predict the future movement of objects, and warn the driver of potential hazards. These ADAS algorithms are constantly being improved as new technologies are being developed. Continuous and consistent advancements in these technologies are making ADASs even more capable of improving road safety and reducing drivers’ workloads.

#### 1.2.3. The Challenges of ADASs

The task of the essential functions of ADASs, namely object detection, recognition, and tracking, is to allow ADASs to identify and track objects in the vehicle’s surroundings, such as other vehicles, pedestrians, cyclists, and sometimes random objects and obstacles, using which ADASs can prevent accidents, keep the vehicle in its lane, and provide other driver assistance features. However, there are various challenges associated with object detection, recognition, and tracking in ADASs, such as:

- Varying environmental conditions: ADASs must be able to operate in a variety of environmental conditions, including different lighting conditions like bright sunlight, dark shadows, fog, daytime, nighttime, etc., different weather conditions such as drizzle, rain, snow, and so on, along with various road conditions including dirt, gravel, etc.;
- Occlusion: objects on the road in real scenarios can often be occluded by other objects, such as other vehicles, pedestrians, or trees, making it difficult for ADASs to detect and track objects;
- Deformation: objects on the road can often be deformed, such as when a vehicle is turning or when a pedestrian is walking, causing difficulties for ADASs in detecting and tracking objects;
- Scale: objects on the road can vary greatly in size, from small pedestrians to large trucks, inducing difficulties for ADASs in detecting and tracking objects of all sizes;
- Multi-object tracking: ADASs must be able to track multiple objects simultaneously, and this can be challenging as objects move and interact with each other in complex ways in real-world scenarios;
- Real-time performance: most importantly, ADASs must be able to detect, recognize, and track objects in real time, which is essential for safety-critical applications, as delays in detection or tracking can lead to accidents and make them unreliable.

Researchers are working on developing newer algorithms and improving the existing algorithms and techniques to address these challenges. Due to this, ADASs are becoming increasingly capable of detecting and tracking objects in a variety of challenging conditions.

#### 1.2.4. The Essentials of ADASs

The above section discusses the challenges of different ADAS methods, whereas in this section, we discuss the numerous requirements of [14,15] ADASs, which must be tackled before the aforementioned issues can be resolved. In other words, ADAS algorithms are facing numerous additional predicaments while working on overcoming the challenges discussed in the previous section:

- The need for accurate sensors: ADASs rely on a variety of sensors to detect and track objects on the road. These sensors must be accurate and reliable to provide accurate information to the ADAS. Nevertheless, sensors are usually affected by factors such as weather, lighting, and the environment, causing difficulties for sensors in providing accurate information, and thus leading to errors in the ADASs;
- The need for reliable algorithms: ADASs also rely on a variety of algorithms to process the data from the sensors and make decisions about how to respond to objects on the road. These algorithms must be reliable to make accurate and timely decisions. However, these algorithms can also be affected by factors such as the complexity of the environment and the number of objects on the road. This makes it difficult for algorithms to make accurate decisions, leading to errors in the ADAS;
- The need for integration with other systems: ADASs must be integrated with different systems in the vehicle, such as the braking system and the steering system. This integration is necessary in order for the ADAS system to take action to avoid probable accidents. Nonetheless, integration is complex and time-consuming, resulting in deployment delays of ADASs;
- The cost of ADASs: ADASs are expensive to develop and deploy, making it difficult for some manufacturers to offer ADASs as standard features in their vehicles. As a result, ADASs are often only available as optional features, which can make them less accessible to all drivers;
- The acceptance of ADASs by drivers: Some drivers may still be hesitant to adopt ADASs because they worry about the technology or they do not trust the technology. This will result in difficulties persuading drivers to opt for vehicles with ADASs.

Despite these challenges, ADASs have the potential to significantly improve road safety. As the technology continues to improve, ADASs are likely to become more affordable and more widely accepted by drivers. This will help to make roads safer for everyone.

### 1.3. ADAS Algorithms: Traditional vs. Deep Learning

There are two main types of algorithms used in ADASs: traditional algorithms and DL algorithms. In this section, we discuss the advantages and disadvantages of traditional and DL algorithms for ADASs and also some of the challenges involved in developing and deploying ADASs.

#### 1.3.1. Traditional Algorithms

Traditional methods for object detection, recognition, and tracking are typically the most common type of algorithms used in ADASs, based on hand-crafted, rule-based features, and heuristics designed to capture the distinctive characteristics of different objects. That is, a feature for detecting vehicles might be the presence of four wheels and a windshield. This means that these algorithms use a set of pre-defined rules to determine what objects are present in the environment and how to respond to them. For instance, a traditional lane-keeping algorithm might use a rule that says, ‘If the vehicle is drifting out of its lane, then turn the steering wheel in the opposite direction’ or ‘a rule might state that if a vehicle is detected in the vehicle’s blind spot, then the driver should be warned’.

Traditional methods are less complex than DL algorithms, making them easier to develop, and are very effective in certain cases, but they are difficult to generalize to new objects or situations because they are limited by the rules that are hard-coded into them. If a new object, obstacle, or hazard is not covered by a rule, then the algorithm may not be able to detect it. Some of the basic traditional methods-based algorithms are:

- Object detection: Traditional object detection algorithms typically use a two-step approach:
  - The region proposal step identifies potential regions in an image that may contain objects, which is typically carried out by using a sliding window approach, where a small window is moved across the image and features are extracted from each window;
  - The classification step classifies each region as an object or background. This is typically carried out by using a machine learning (ML) algorithm, such as a support vector machine (SVM) [16] or a random forest [17];
- Object recognition: Traditional object recognition algorithms typically use a feature-based approach:
  - The feature extraction step extracts features from an image that are relevant to the object class, which is typically carried out by using hand-crafted features, such as color histograms [18], edge features [19], or shape features [20];
  - The classification step classifies the object class by using a ML algorithm, such as a SVM [16] or random forest [17];
- Object tracking: Traditional object-tracking algorithms typically use a Kalman filter [21]:
  - The state estimation step estimates the state of the object, such as its position, velocity, and acceleration;
  - The measurement update step updates the state estimate based on new measurements of the object.

These traditional object detection, recognition, and tracking algorithm are effective for a variety of ADAS applications. However, they can be computationally expensive and may not be able to handle challenging conditions, such as occlusion or low lighting.

In recent years, there has been a trend towards using DL algorithms for object detection, recognition, and tracking in ADASs. DL algorithms have been shown to be more accurate than traditional algorithms, and they can handle challenging conditions more effectively.

#### 1.3.2. Deep Learning Algorithms

Inspired by the human brain, DL methods for object detection, recognition, and tracking use artificial neural networks (ANNs) to learn the features that are important for identifying different objects. They are composed of layers of interconnected nodes. Each node performs a simple calculation, and the output of each node is used as the input to the next node.

DL algorithms can learn to detect objects, obstacles, and hazards from large datasets of labeled data usually collected using a variety of sensors. The algorithm is trained to associate specific patterns in the data with specific objects or hazards. DL algorithms are generally more complex than traditional algorithms, but they can achieve higher accuracy as they are not limited by hand-crafted rules, they can learn to detect objects and hazards not covered by any rules, and they are also able to handle challenging conditions, such as occlusion or low lighting, more effectively. Some of the standard DL method-based algorithms are discussed below:

- Object detection: DL object detection algorithms commonly use a convolutional neural network (CNN) to extract features from an image. The CNN is then trained on a dataset of images that have been labeled with the objects that they contain. Once the CNN is trained, it can be used to detect objects in new images;
- Object recognition: DL object recognition algorithms also conventionally use a CNN to extract features from an image. However, the CNN is trained on a dataset of images that have been labeled with the class of each object. The trained CNN can be used to recognize the class of objects in new images;
- Object tracking: DL object tracking algorithms typically use a combination of CNNs and Kalman filters [21]. The CNN is used to extract features from an image and the Kalman filter is used to track the state of the object over time.

## 2. Sensors Used in Object Detection, Recognition, and Tracking Algorithms of ADASs

Several sensors can be used for object detection, recognition, and tracking in ADASs. The most common sensors include cameras, radars, and lidars. In addition to these sensors, some other sensors can also be used, such as:

- Ultrasonic sensors: used to detect objects that are close to the vehicle, aiding in preventing collisions with pedestrians or other vehicles;
- Inertial measurement units (IMUs): employed to track the movement of the vehicle using which the accuracy of object detection and tracking can be improved;
- GPS sensors: used to determine the position of the vehicle and are utilized to track the movement of the vehicle and to identify objects that are in the vehicle’s path;
- Gyroscope sensors: used to track the orientation of the vehicle and employed to improve the accuracy of object detection and tracking algorithms.

The choice of sensors for object detection, recognition, and tracking in ADASs depends on the specific application. For instance, a system that is designed to detect pedestrians may use a combination of cameras and radar, while a system that is designed to track the movement of other vehicles may use a combination of radar and lidar.

The combination of multiple sensors is mostly used in more recent state-of-the-art methods, as this improves the accuracy of object detection, recognition, and tracking algorithms. The combination of sensors combines the strengths of the sensors and overcomes the weaknesses of the other sensors.

### 2.1. Cameras, Radar, and Lidar

Cameras, radar, and lidar are the most common types of sensors used in ADASs. While there are two main types of cameras—monocular cameras are the most common type used in ADASs, which have a single lens and can only see in two dimensions, while stereo cameras have two lenses and can see in three dimensions—there are no distinctive types of radars and lidars. These sensors are used in ADASs in a variety of ways, including:

- Object detection: the sensors are used to detect objects in the road environment such as pedestrians, vehicles, cyclists, and traffic signs, and then warn the driver of potential hazards or take corrective actions like braking or steering control using the gathered information;
- Object recognition: the sensors are used to recognize the class of an object, such as a pedestrian, a vehicle, a cyclist, or a traffic sign. This information can be used to provide the driver with more information about the hazard, such as the type of vehicle, the type of traffic sign and the road condition ahead, or the speed of a pedestrian;
- Object tracking: the sensors can be used to track the movement of an object over time, which is then used to predict the future position of an object, which can be used to warn the driver of potential collisions.

The advantages of cameras are their low cost, ease of installation, wide field of view (FOV), and high resolution, but they are easily impacted by weather conditions, occlusion of objects, and varying light conditions. On the other hand, both radars and lidars are resistant to varying weather conditions such as rain, snow, fog, and so on. While radars are occlusion-resistant and provide a longer range than cameras, they fail to provide as many details as cameras and are more expensive than cameras. Compared to both cameras and radars, lidars provide very accurate information about the distance and shape of objects, even in difficult conditions, and possess 3D capabilities, enabling them to create a 3D map of the road environment that makes it easier and more efficient to identify and track objects that are occluded by other objects. Nonetheless, lidars are more expensive than cameras and radars, and lidar systems are more complex, making them more challenging to install and maintain. Cameras are used in almost all ADAS functions, while radars and lidars are used in FCWS, LDWS, BSD, and ACC, with lidars having an additional application in autonomous driving.

All the above features allow these versatile sensors to be used for a variety of object detection, recognition, and tracking tasks in ADASs. However, some challenges need to be addressed before they can be used effectively in all conditions. Hence, some researchers have attempted to use a combination of these sensors, as discussed in the following section.

### 2.2. Sensor Fusion

Sensor fusion is the process of combining data from multiple sensors to create a more complete and accurate picture of the world. This can be used to improve the performance of object detection, recognition, and tracking algorithms in ADASs.

Numerous different sensor fusion techniques can be used for ADASs, namely:

- Data-level fusion: a technique that combines data from different sensors at the data level by averaging the data from different sensors, or by using more sophisticated techniques such as Kalman filtering [21,22];
- Feature-level fusion: combines features extracted from data from different sensors by combining the features, or by using more sophisticated techniques such as Bayesian fusion [23,24];
- Decision-level fusion: combines decisions made by different sensors by taking the majority vote, or by using more sophisticated techniques such as the Dempster–Shafer theory [25,26,27].

The choice of sensor fusion technique is application-specific. A data-level fusion may be a good choice for applications where accuracy is critical, whereas a decision-level fusion may be a good choice for applications where speed is critical.

The benefits of using sensor fusion for object detection, recognition, and tracking in ADASs can be listed as [15,28,29,30,31]:

- Improved accuracy: sensor fusion improves the accuracy of object detection, recognition, and tracking algorithms by combining the strengths of different sensors;
- Improved robustness: sensor fusion also improves the robustness of object detection, recognition, and tracking algorithms by making them less susceptible to noise and other disturbances;
- Reduced computational complexity: sensor fusion also reduces the computational complexity of object detection, recognition, and tracking algorithms, as the data from multiple sensors can be processed together, resulting in saved time and processing power.

Overall, sensor fusion is a promising, powerful technique that has the potential to make ADAS object detection, recognition, and tracking algorithms much safer and more reliable. Although sensor fusion is advantageous, it has some challenges [15,32], such as:

- Data compatibility: the data from different sensors must be compatible to be fused, implying the data must be in the same format and have the same resolution;
- Sensor calibration: the sensors must be calibrated to ensure that they are providing accurate data, which can be challenging, especially if the sensors are in motion;
- Computational complexity: Sensor fusion is computationally expensive, especially if a large number of sensors are being fused. This can limit the use of sensor fusion in real-time applications.

Despite these challenges, sensor fusion is emerging with greater potential to improve the performance of ADAS object detection, recognition, and tracking algorithms. As sensor technology continues to improve, a fusion of sensors will become even more powerful and efficient, and it will likely become a standard feature in ADASs.

The following section discusses the most commonly fused sensors in ADASs.

#### 2.2.1. Camera–Radar Fusion

Camera–radar fusion is a technique that combines data from cameras and radar sensors to improve the performance of object detection, recognition, and tracking algorithms in ADASs. As cameras are good at providing good image quality but are susceptible to weather conditions, radar sensors compensate by seeing through weather conditions. Data-level fusion and decision-level fusion are the two main approaches to camera–radar fusion.

#### 2.2.2. Camera–Lidar Fusion

Camera–lidar fusion is a technique that combines data from cameras and lidar sensors to improve the performance of object detection, recognition, and tracking algorithms in ADASs. Cameras are good at providing detailed information about the appearance of objects, while lidar sensors are good at providing information about the distance and shape of objects. By combining data from these two sensors, it is feasible to create a complete and accurate picture of the object, leading to improved accuracy in object detection and tracking.

#### 2.2.3. Radar–Lidar Fusion

Radar–lidar fusion is a technique that combines the data from radar and lidar sensors, improving the performance of ADAS algorithms. Radar sensors use radio waves to detect objects at long distances, while lidar sensors use lasers to detect objects in detail. By fusing the data from the two sensors, the system can obtain a more complete and accurate view of the environment.

#### 2.2.4. Lidar–Lidar Fusion

Lidar–lidar fusion is a technique that combines data from two or more lidar sensors, improving the performance of object detection, recognition, and tracking algorithms in ADASs. Lidar sensors are good at providing information about the distance and shape of objects, but they can be limited in their ability to detect objects that are close to the vehicle or that are occluded by other objects. By fusing data from multiple lidar sensors, it is possible to create a complete and accurate picture of the environment, which can lead to improved accuracy in object detection and tracking.The above discussed advantages and disadvantages of various ADASs sensors are listed in the Table 1.

## 3. Systematic Literature Review

The main objective of this review is to determine the latest trends and approaches implemented for different ADAS methods in autonomous vehicles and discuss their achievements. This paper also attempts to evaluate the valuable basis of the methods, implementation, and applications to furnish a state-of-the-art understanding for new researchers in this computer vision and autonomous vehicles field. 

The writing of this paper follows a planned, conducted, and observed process. The planning phase involved clarifying the research questions and review protocol, which comprised identifying the publications’ sources, keywords to search for, and selection criteria. The conducting phase involved analyzing, extracting, and synthesizing the literature collection. This included identifying the key themes and findings from the literature and drawing conclusions that address the research questions and objectives. The observed stage contained the review results, addressing the summary of the key findings as well as any limitations or implications of the study.

### 3.1. Research Questions (RQs)

The main objective of this review is to determine the trend of the methods implemented for different ADAS methods in the field of autonomous vehicles, as well as the achievements of the latest techniques. Additionally, we aim to provide a valuable foundation for the methods, challenges, and opportunities, thus providing state-of-the-art knowledge to support new research in the field of computer vision and ADASs.

Two research questions (RQs) have been defined as follows:

1. What techniques have been implemented for different ADAS methods in an autonomous vehicle?
2. What dataset was applied for the network training, validation, and testing?

A focused approach has been adopted while scanning the literature. First, each article was reviewed to see if it answered the earlier questions. The information acquired was then presented comprehensively to achieve the vision of this article.

### 3.2. Review Protocol

Below, we have listed the literature search sources, search terms, and inclusion and exclusion selection criteria, as well as the technique of literature collection used for this systematic literature review (SLR).

#### 3.2.1. Search Sources

IEEE Xplore and MDPI were chosen as the databases from which the data were extracted.

#### 3.2.2. Search Terms

Different sets of search terms were used to investigate the various ADAS methods presented in this research. The OR, AND, and NOT operators were used to select and combine the most relevant and commonly used applicable phrases. The AND operator combined individual search strings into a search query. The databases included IEEE Xplore and MDPI. The search terms used for the respective different methods of ADASs are listed in the respective sections of this paper.

#### 3.2.3. Inclusion Criteria

The study covered all primary publications published in English that discussed the different ADAS methods or any other task related to them discussed in this paper. There were no constraints on subject categories or time frames for a broad search spectrum. The selected articles were among the top most cited journal papers published across four years, from 2019 to 2022.

In addition, the below parameters were also considered while selecting the papers:

- Relevance of the research papers to the topic of the review paper covering the most important aspects of the topic and providing a comprehensive overview of the current state of knowledge;
- The quality of the research papers should be high. They should be well written, well argued, and well supported by implementation details and experimental results;
- Coverage of the research papers should include a wide range of perspectives on the topic and not limited to a single viewpoint or approach;
- The methodology presented in the research papers should be sound such that the research methods must be rigorous and provide clear evidence to support their conclusions;
- The research papers should be well written and easy to understand in a clear and concise style so that the information is accessible and understandable to a wide audience;
- The research papers should have had a significant impact on the field. They should have been cited by other researchers and used to inform new research.

#### 3.2.4. Exclusion Criteria

Articles written in languages other than English were not considered. The exclusion criteria also included short papers, such as abstracts or expanded abstracts, earlier published versions of the detailed works, and survey/review papers.

## 4. Discussion—Methodology

### 4.1. Vehicle Detection

Vehicle detection, one of the key components and a critical task of ADASs, is the process of identifying and locating vehicles in the surrounding scenes using sensors such as cameras, radars, and lidar employing computer vision techniques. This information is used to provide drivers with warnings about potential hazards, such as cars that are too close or that are changing lanes and pedestrians or cyclists that might be in the vehicles’ way. It is a crucial function for many ADAS features, such as ACC, LDWS, FCWS, and BSD, discussed in the later sections of the paper. 

Vehicle detection is a challenging task, as vehicles vary in size, shape, and color, affecting their appearance in images and videos. They can be seen from a variety of different angles, which can also affect their appearance; furthermore, vehicle sizes can be too small or too big, they could be partially or fully occluded by other objects in the scene; there are different types of vehicles, each with a unique appearance, and the lighting conditions and possible background clutter also affect the appearance of vehicles. All of these factors make detection challenging.

Despite these challenges, the vehicle detection algorithm in ADASs has greatly evolved and is still evolving, and there have been significant advances in vehicle detection over the years. Early algorithms were based on relatively simple-to-implement image processing techniques, such as edge detection and color segmentation, but they were not very accurate. In the early 2000s, there was a shift towards using ML techniques that can learn from data, making them more accurate than simple image processing techniques. Some of the most common ML algorithms used for vehicle detection include support vector machines (SVMs), random forests, and DL NNs.

Deep learning NNs are the most effective machine learning algorithms for vehicle detection. Deep learning NNs can learn complex features from data, which makes them very accurate. Regardless, DL NNs are also more computationally expensive than other ML algorithms. In recent years, there has been a trend towards using sensor fusion for vehicle detection.

The vehicle detection algorithms in ADASs are still evolving. As sensor technology continues to improve, and as ML algorithms become more powerful, vehicle detection algorithms will become even more accurate and reliable.

#### Search Terms and Recent Trends in Vehicle Detection

**Figure 2:**
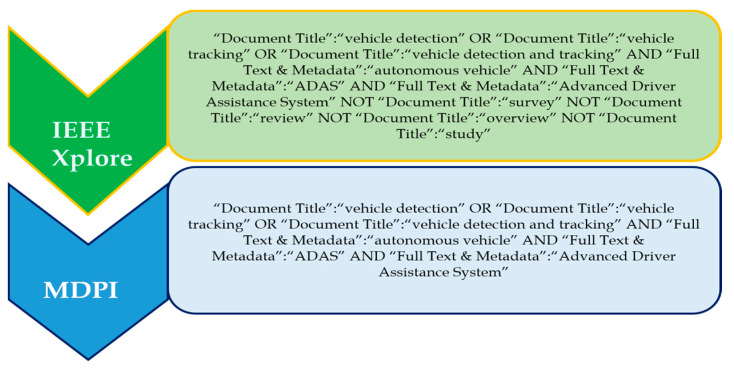
Search queries for each of the databases for vehicle detection. The databases include IEEE Xplore and MDPI.

‘Vehicle detection’, ‘vehicle tracking’, and ‘vehicle detection and tracking’ are three prominent search terms which were used to investigate the topic. The ‘OR’ operator was used to choose and combine the most relevant and regularly used applicable phrases; that is, the search phrases ‘vehicle detection’, ‘vehicle tracking’, and ‘vehicle detection and tracking’ were discovered. Figure 2 shows the complete search query for each of the databases. The databases include IEEE Xplore and MDPI.

Since the evolution of vehicle detection has been rapid, considering the detection, recognition, and tracking of other vehicles, pedestrians, and objects, plenty of different methods have been proposed in the past few years. Some of the recent prominent state-of-the-art vehicle detection methods are discussed in the following sections.

Ref. [33] presents a scale-insensitive CNN, SINet, which is designed for rapid and accurate vehicle detection. SINet employs two lightweight techniques: context-aware RoI pooling and multi-branch decision networks. These preserve small-scale object information and enhance classification accuracy. Ref. [34] introduces an integrated approach to monocular 3D vehicle detection and tracking. It utilizes a CNN for vehicle detection and employs a Kalman filter-based tracker for temporal continuity. The method incorporates multi-task learning, 3D proposal generation, and Kalman filter-based tracking. Combining radar and vision sensors, ref. [35] proposes a novel distant vehicle detection approach. Radar generates candidate bounding boxes for distant vehicles, which are classified using vision-based methods, ensuring accurate detection and localization. Ref. [36] focuses on multi-vehicle tracking, utilizing object detection and viewpoint estimation sensors. The CNN detects vehicles, while viewpoint estimation enhances tracking accuracy. Ref. [37] utilizes CNN with feature concatenation for urban vehicle detection, improving robustness through layer-wise feature combination. Ref. [38] presents a robust vehicle detection and counting method integrating CNN and optical flow, while [39] pioneers vehicle detection and classification via distributed fiber optic acoustic sensing. Ref. [40] introduces a vehicle tracking and speed estimation method using roadside lidar, incorporating a Kalman filter. Ref. [41] modifies Tiny-YOLOv3 for front vehicle detection with SPP-Net enhancement, excelling in challenging conditions. Ref. [42] proposes an Extended Kalman Filter (EKF) for vehicle tracking using radar and lidar data, while [43] enhances SSD for accurate front vehicle detection. Ref. [44] improves Faster RCNN for oriented vehicle detection in aerial images with feature amplification and oversampling. Ref. [45] employs reinforcement learning with partial vehicle detection for efficient intelligent traffic signal control. Ref. [46] presents a robust DL framework for vehicle detection in adverse weather conditions. Ref. [47] adopts GAN-based image style transfer for nighttime vehicle detection, while ref. [48] introduces MultEYE for real-time vehicle detection and tracking using UAV imagery. Ref. [49] analyzes traffic patterns during COVID-19 using Planet remote-sensing satellite images for vehicle detection. Ref. [50] proposes one-stage anchor-free 3D vehicle detection from lidar, ref. [51] fuses RGB-infrared images for accurate vehicle detection using uncertainty-aware learning. Ref. [52] optimizes YOLOv4 for improved vehicle detection and classification. Ref. [53] introduces a real-time foveal classifier-based system for nighttime vehicle detection. Ref. [54] combines YOLOv4 and SPP-Net for multi-scale vehicle detection in varying weather. Ref. [55] efficiently detects moving vehicles with a CNN-based method incorporating background subtraction. Ref. [56] refines YOLOv5 for vehicle detection in aerial infrared images, ensuring robustness against challenges like occlusion and low contrast.

Overall, the aforementioned papers represent a diverse set of approaches to vehicle detection and tracking. Each paper has its strengths and weaknesses, and it is important to consider the specific application when choosing a method. However, all of the papers represent significant advances in the field of vehicle detection and tracking. The list of reviewed papers on vehicle detection is summarized in Table 2.

### 4.2. Pedestrian Detection

Pedestrian detection is also a key component of ADASs that uses sensors to identify and track pedestrians in the surrounding environment and prevent collisions with pedestrians. The goal of pedestrian detection is to identify and track pedestrians in the surrounding environment, warn the driver of potential collisions with pedestrians, and take evasive action such as automatically applying brakes, if necessary.

Pedestrian detection systems typically use a combination of sensors, such as cameras, radar, and lidar. Cameras are often used to identify the shape and movement of pedestrians, while radar and lidar can be used to determine the distance and speed of pedestrians. Cameras can be susceptible to glare and shadows, whereas radar and lidars are less susceptible to these problems.

Pedestrian detection systems can be used to warn drivers of potential collisions in a variety of ways. Some systems simply alert the driver with a visual or audible warning. Others can take more active measures, such as automatically braking the vehicle or slightly steering it away from the pedestrian. However, pedestrian detection is more challenging, as pedestrians are often smaller and more difficult to distinguish from other objects in the environment. Thus, it is an important safety feature for ADASs, as it can help to prevent accidents involving pedestrians. According to the National Highway Traffic Safety Administration (NHTSA) [57], pedestrians are involved in about 17% of all traffic fatalities in the United States. Pedestrian detection systems can help to reduce this number by warning drivers of potential hazards and by automatically applying the brakes in emergencies.

#### Search Terms and Recent Trends in Pedestrian Detection

**Figure 3:**
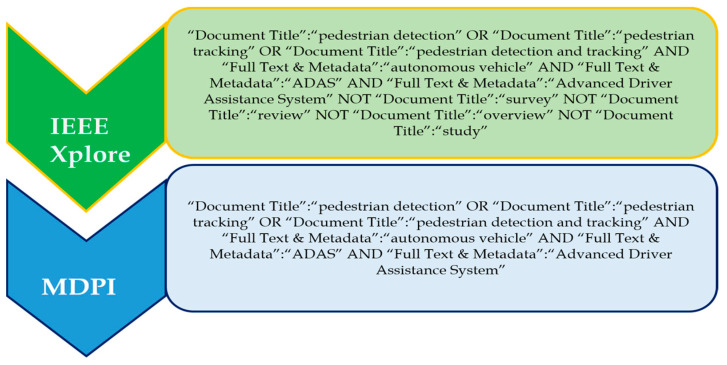
Search queries for each of the databases for pedestrian detection. The databases include IEEE Xplore and MDPI.

‘Pedestrian detection’, ‘pedestrian tracking’, and ‘pedestrian detection and tracking’ are three prominent search terms which were used to investigate this topic. The ‘OR’ operator was used to choose and combine the most relevant and regularly used applicable phrases; that is, the search phrases pedestrian detection, ‘pedestrian tracking’, and ‘pedestrian detection and tracking’ were discovered. Figure 3 shows the complete search query for each of the databases. The databases include IEEE Xplore and MDPI.

Ref. [58] introduces a novel approach to pedestrian detection, emphasizing high-level semantic features instead of traditional low-level features. This method employs context-aware RoI pooling and a multi-branch decision network to preserve small-scale object details and enhance classification accuracy. The CNN initially captures high-level semantic features from images, which are then used to train a classifier to distinguish pedestrians from other objects. Ref. [59] proposes an adaptive non-maximum suppression (NMS) technique tailored for refining pedestrian detection in crowded scenarios. Conventional NMS algorithms often eliminate valid detections along with duplicates in crowded scenes. The new ‘Adaptive NMS’ algorithm dynamically adjusts the NMS threshold based on crowd density, enabling the retention of more pedestrian candidates in congested areas. Ref. [60] introduces the ‘Mask-Guided Attention Network’ (MGAN) for detecting occluded pedestrians. Utilizing a CNN, MGAN extracts features from both pedestrians and backgrounds. Pedestrian features guide the network’s focus towards occluded regions, improving the accuracy of detecting occluded pedestrians. Ref. [61] presents a real-time method to track pedestrians by utilizing camera and lidar sensors in a moving vehicle. Combining sensor features enables accurate pedestrian tracking. Features from the camera image, such as silhouette, clothing, and gait, are extracted. Additionally, features like height, width, and depth are obtained from the lidar point cloud. These details facilitate precise tracking of pedestrians’ locations and poses over time. A Kalman filter enhances tracking performance through sensor data fusion, offering better insights into pedestrian behavior in dynamic environments. Ref. [62] proposes a computationally efficient single-template matching technique for accurate pedestrian detection in lidar point clouds. The method creates a pedestrian template from training data and uses it to identify pedestrians in new point clouds, even under partial occlusion. Ref. [63] focuses on tracking pedestrian flow and statistics using a monocular camera and a CNN–Kalman filter fusion. The CNN extracts features from the camera image, which is followed by a Kalman filter for trajectory estimation. This approach effectively tracks pedestrian flow and vital statistics, including count, speed, and direction.

Ref. [64] addresses hazy weather pedestrian detection with deep learning. DL models are trained on hazy weather datasets and use architectural modifications to handle challenging conditions. This approach achieves high pedestrian detection accuracy, even in hazy weather. Ref. [65] introduces the ‘NMS by Representative Region’ algorithm to refine pedestrian detection in crowded scenes. By employing representative regions, this method enhances crowded scene handling by comparing these regions and removing duplicate detections, resulting in reduced false positives. Ref. [66] proposes a graininess-aware deep feature learning approach, equipping DL models to handle grainy images. A DL model is trained using a graininess-aware loss function on a dataset containing grainy and non-grainy pedestrian images. This model effectively detects pedestrians in new images, even when they are grainy. Ref. [67] presents a DL framework for real-time vehicle and pedestrian detection on rural roads, optimized for embedded GPUs. Modified Faster R-CNN detects both vehicles and pedestrians simultaneously in rural road scenes. A new rural road image dataset is developed for training the model. Ref. [68] addresses infrared pedestrian detection at night using an attention-guided encoder–decoder CNN. Attention mechanisms focus on relevant regions in infrared images, enhancing detection accuracy in low-light conditions. Ref. [69] focuses on improved YOLOv3-based pedestrian detection in complex scenarios, incorporating modifications to handle various challenges like occlusions, lighting variations, and crowded environments.

Ref. [70] introduces Ratio-and-Scale-Aware YOLO (RASYOLO), handling pedestrians with varying sizes and occlusions through ratio-aware anchors and scale-aware feature fusion. Ref. [71] introduces Track Management and Occlusion Handling (TMOH), managing occlusions and multiple-pedestrian tracking through track suspension and resumption. Ref. [72] incorporates a Part-Aware Multi-Scale fully convolutional network (PAM-FCN) to enhance pedestrian detection accuracy by considering pedestrian body part information and addressing scale variation. Ref. [73] proposes Attention Fusion for One-Stage Multispectral Pedestrian Detection (AFOS-MSPD), combining attention fusion and a one-stage approach for multispectral pedestrian detection, improving efficiency and accuracy. Ref. [74] utilizes multispectral images for Multispectral Pedestrian Detection (MSPD), improving detection using a DNN designed for multispectral data. Ref. [75] presents Robust Pedestrian Detection Based on Multi-Spectral Image Fusion and Convolutional Neural Networks (RPOD-FCN), utilizing multi-spectral image fusion and a CNN-based model for accurate detection.

Ref. [76] introduces Uncertainty-Guided Cross-Modal Learning for Robust Multispectral Pedestrian Detection (UCM-RMPD), addressing multispectral detection challenges using uncertainty-guided cross-modal learning. Ref. [77] focuses on multimodal pedestrian detection for autonomous driving using a Spatio-Contextual Deep Network-Based Multimodal Pedestrian Detection (SCDN-PMD) approach. Ref. [78] proposes a Novel Approach to Model-Based Pedestrian Tracking Using Automotive Radar (NMPT radar), utilizing radar data for model-based pedestrian tracking. Ref. [79] adopts YOLOv4 Architecture for Low-Latency Multispectral Pedestrian Detection in Autonomous Driving (AYOLOv4), enhancing detection accuracy using multispectral images. Ref. [80] introduces modifications to [79] called AIR-YOLOv3, an improved network-pruned YOLOv3 for aerial infrared pedestrian detection, enhancing robustness and efficiency. Ref. [81] presents YOLOv5-AC, an attention mechanism-based lightweight YOLOv5 variant for efficient pedestrian detection on embedded devices. The list of reviewed papers on pedestrian detection is summarized in Table 3.

### 4.3. Traffic Signs Detection

Traffic Signs Detection and Recognition (TSR) is another key component of ADASs that automatically detects and recognizes traffic signs on the road and provides information to the driver regarding speed limits, upcoming turns, and so on. TSR systems typically use cameras to capture images of traffic signs and then use computer vision algorithms to identify and classify the signs.

TSR systems can be a valuable safety feature, as they can help to prevent accidents caused by driver distraction or drowsiness. For example, TSR systems can alert drivers to speed limit changes, stop signs, and yield signs. They can also help drivers to stay in their lane and avoid crossing over into oncoming traffic. Although TSR can be challenging due to the variety of traffic signs, the different fonts and styles used, and the presence of noise and clutter, TSR systems are becoming increasingly common in new vehicles. The NHTSA has mandated that all new cars sold in the United States come equipped with TSR systems by 2023 [57].

#### Search Terms and Recent Trends in Traffic Signs Detection

**Figure 4:**
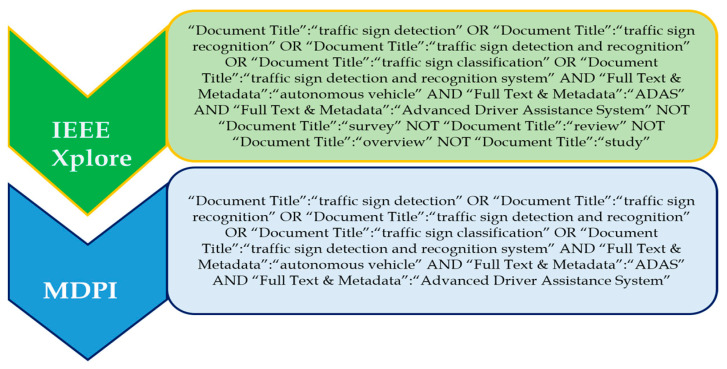
Search queries for each of the databases for traffic sign detection. The databases include IEEE Xplore and MDPI.

‘Traffic sign detection’, ‘traffic sign recognition, ‘traffic sign classification’, ‘traffic sign detection and recognition’, and ‘traffic sign detection and recognition system’ are some of the prominent search terms which were used to investigate this topic. The ‘OR’ operator was used to choose and combine the most relevant and regularly used applicable phrases; that is, the search phrases ‘driver monitoring system’ and ‘driver monitoring and assistance system’ were discovered. Figure 4 shows the complete search query for each of the databases. The databases include IEEE Xplore and MDPI.

Yuan et al. [82] introduce VSSA-NET, a novel architecture for traffic sign detection (TSD), which employs a vertical spatial sequence attention network to improve accuracy in complex scenes. VSSA-NET extracts features via CNN, followed by a vertical spatial sequence attention module to emphasize vertical locations crucial for TSD. The detection module outputs traffic sign bounding boxes. Li and Wang [83] present real-time traffic sign recognition using efficient CNNs, addressing diverse lighting and environmental conditions. MobileNet extracts features from input images, followed by SVM classification. Liu et al. [84] propose multi-scale region-based CNN (MR-CNN) for recognizing small traffic signs. MR-CNN extracts multi-scale features using CNN, generates proposals with RPN, and uses Fast R-CNN for classification and bounding box outputs. Tian et al. [85] introduce a multi-scale recurrent attention network for TSD. CNN extracts multi-scale features, the recurrent attention module prioritizes scale, and the detection module outputs bounding boxes for robust detection across scenarios. Cao et al. [86] present improved TSDR for intelligent vehicles. CNN performs feature extraction, RPN generates region proposals, and SVM classifies proposals, enhancing reliability in dynamic road environments. Shao et al. [87] improve Faster R-CNN TSD with a second RoI and HPRPN. CNN performs feature extraction, RPN generates region proposals, and the second RoI refines proposals, enhancing accuracy in complex scenarios.

Zhang et al. [88] propose cascaded R-CNN with multiscale attention for TSD. RPN generates proposals, Fast R-CNN classifies, and multiscale attention improves detection performance, particularly when there is an imbalanced data distribution. Tabernik and Skočaj [89] explore the DL framework for large-scale TSDR. CNN performs feature extraction, RPN generates region proposals, and Fast R-CNN classifies, exploring DL’s potential in handling diverse real-world scenarios. Kamal et al. [90] introduce automatic TSDR using SegU-Net and modified Tversky loss. SegU-Net segments traffic signs and modified loss function enhances detection and recognition, handling appearance variations. Tai et al. [91] propose a DL approach for TSR with spatial pyramid pooling and scale analysis. CNN performs feature extraction, while spatial pyramid pooling captures context and scales, enhancing recognition across scenarios. Dewi et al. [92] evaluate the spatial pyramid pooling technique on CNN for TSR system robustness. Assessing pooling sizes and strategies, they evaluate different CNN architectures for effective traffic sign recognition. Nartey et al. [93] propose robust semi-supervised TSR with self-training and weakly supervised learning. CNN performs feature extraction, self-training labels unlabeled data, and weakly supervised learning classifies labeled data, enhancing accuracy using limited labeled data. 

Dewi et al. [94] leverage YOLOv4 with synthetic GAN-generated data for advanced TSR. YOLOv4 with synthetic data from BigGAN achieves top performance, enhancing detection on the GTSDB dataset. Wang et al. [95] improve YOLOv4-Tiny TSR with new features and classification modules. New data augmentation improves the performance on the GTSDB dataset, optimizing recognition while maintaining efficiency. Cao et al. [96] present improved sparse R-CNN for TSD with a new RPN and loss function. Enhancing detection accuracy using advanced techniques within the sparse R-CNN framework. Lopez-Montiel et al. [97] propose DL-based embedded system evaluation and synthetic data generation for TSD. Methods to assess DL system performance and efficiency for real-time TSD applications are developed. Zhou et al. [98] introduce a learning region-based attention network for TSR. The attention module emphasizes important image regions, potentially enhancing recognition accuracy. Koh et al. [99] evaluate senior adults’ TSR recognition through EEG signals, utilizing EEG signals to gain unique insights into senior individuals’ traffic sign perception.

Ahmed et al. [100] present a weather-adaptive DL framework for robust TSR. A cascaded detector with a weather classifier improves TSD performance in adverse conditions, enhancing road safety. Xie et al. [101] explore efficient federated learning in TSR with spike NNs (SNNs). SNNs enable training on decentralized datasets, minimizing communication overhead and resources. Min et al. [102] propose semantic scene understanding and structural location for TSR, leveraging scene context and structural information for accurate traffic sign recognition. Gu and Si [103] introduce a lightweight real-time TSD integration framework based on YOLOv4. Novel data augmentation and YOLOv4 optimization are used for speed and accuracy, achieving real-time performance. Liu et al. [104] introduce the M-YOLO TSD algorithm for complex scenarios. M-YOLO detects and classifies traffic signs, addressing detection in intricate environments. Wang et al. [105] propose real-time multi-scale TSD for driverless cars. The multi-scale approach detects traffic signs of various sizes, enhancing performance in diverse scenarios. The list of reviewed papers on traffic signs detection is summarized in Table 4.

### 4.4. Driver Monitoring System (DMS)

A driver monitoring system (DMS), also called a driver monitoring and assistance system (DMAS), is a camera-based safety system used to assess the driver’s alertness and attention. It monitors a driver’s behavior by detecting and tracking the driver’s face, eyes, and head position and warns or alerts them when they become distracted or drowsy for long enough to lose situational awareness or full attention to the task of driving. DMSs can also use other sensors, such as radar or infrared sensors, to gather additional information about the driver’s state.

DMSs are becoming increasingly common in vehicles and are used to monitor the driver’s alertness and attention. This information is then used to prevent accidents and save lives by warning the driver if they are starting to become drowsy or distracted. Some of the latest DMSs can even predict if drivers are eating and drinking while driving.

#### 4.4.1. Driver Monitoring System Methods

There are a variety of methods used in DMSs. One common approach is to use a camera to monitor the driver’s face, while the other approach is to use a sensor fusion approach, which combines data from multiple sensors, such as cameras, radar, and eye tracking sensors.

DMSs can use a variety of sensors to monitor the driver, including:

- Facial recognition. This is the most common type of sensor used in DMSs. Facial recognition systems can track the driver’s face and identify signs of distraction or drowsiness, such as eye closure, head tilt, and lack of facial expression.
- A head pose sensor tracks the position of the driver’s head and can identify signs of distraction or drowsiness, such as looking away from the road or nodding off.
- An eye gaze sensor tracks the direction of the driver’s eye gaze and can identify signs of distraction or drowsiness, such as looking at the phone or dashboard.
- An eye blink rate sensor tracks the driver’s eye blink rate and can identify signs of drowsiness, such as a decrease in the blink rate.
- Speech recognition is used in DMSs to detect if the driver is talking on the phone or if they are not paying attention to the road.

The above sensors are used in DMSs to detect a variety of driver behaviors, such as (i) when a driver is distracted by looking away from the road, talking on the phone, or using a mobile device; (ii) when a driver is drowsy, which can be determined by tracking the driver’s eye movements and eyelid closure; (iii) when a driver is inattentive, which can be determined by tracking the driver’s head position and eye gaze.

When a DMS detects risky driver behavior, it can provide a variety of alerts to the driver, including alerts displayed on the dashboard or windshield, referred to as visual alerts; alerts played through the vehicle’s speakers, which are called audio alerts; and hectic alerts, in which alerts are issued through vibrations of the steering wheel or the driver’s seat. In some cases, the DMS may also take corrective action, such as applying the brakes or turning off the engine.

#### 4.4.2. Search Terms and Recent Trends in Driver Monitoring System Methods 

**Figure 5:**
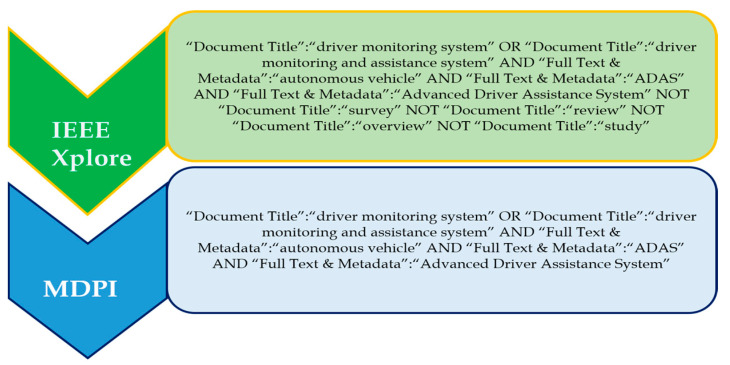
Search queries for each of the databases for the driver monitoring system. The databases include IEEE Xplore and MDPI.

‘Driver monitoring system’ and ‘driver monitoring and assistance system’ are the two prominent search terms used to investigate this topic. The ‘OR’ operator was used to choose and combine the most relevant and regularly used applicable phrases. That is, the search phrases ‘driver monitoring system’ and ‘driver monitoring and assistance system’ were discovered. Figure 5 shows the complete search query for each of the databases. The databases include IEEE Xplore and MDPI.

The papers [106,107,108,109,110,111,112,113,114] discuss a variety of approaches to DMSs. These include some of the key methods like (i) the powerful technique employing DL, which is used to extract features from images and videos. These are used to identify driver behaviors such as eye closure, head pose, and facial expressions. (ii) A more general approach is using machine learning, which can be used to learn patterns from data. These are used to identify driver behaviors that are not easily captured using traditional methods, such as hand gestures and body language, and (iii) a technique that combines data from multiple sensors, referred to as sensor fusion, to improve the accuracy of DMSs. For instance, a DMS could combine data from a camera, an eye tracker, and a heart rate monitor to provide a more comprehensive assessment of the driver’s state.

Y. Zhao et al. [106] propose a novel real-time DMSs based on deep CNN to monitor drivers’ behavior and detect distractions. It uses video input from an in-car camera and employs CNNs to analyze the driver’s facial expressions and head movements to assess their attentiveness. It can detect eye closure, head pose, and facial expressions with high accuracy. Ref. [107] works towards a DMS that uses machine learning to estimate driver situational awareness using eye-tracking data. It aims to predict driver attention and alertness to the road, enhancing road safety. Ref. [108] proposes a lightweight DMS based on Multi-Task Mobilenets architecture, which efficiently monitors drivers’ behavior and attention using low computational resources. It can even run on a simple smartphone, making it suitable for real-time monitoring. Ref. [109] introduces an optimization algorithm for DMSs using DL. This algorithm improves the accuracy of the DMS by reducing the number of false positives and ensuring real-time performance.

Ref. [110] proposes a real-time DMS based on visual cues, leveraging facial expressions and eye movements to assess driver distraction and inattention. It is able to detect driver behaviors such as eye closure, head pose, and facial expressions using only a camera. Ref. [111] proposes an intelligent DMS that uses a combination of sensors and ML. It is capable of providing a comprehensive assessment of the driver’s state, including their attention level, fatigue, and drowsiness, and provides timely alerts to improve safety. Ref. [112] proposes a hybrid DMS combining Internet of Things (IoT) and ML techniques for comprehensive driver monitoring. It collects data from multiple sensors and uses ML to identify driver behaviors. Ref. [113] focuses on a distracted DMS that uses AI to detect and prevent risky behaviors on the road. It detects distracted driving behaviors such as texting and talking on the phone while driving. Ref. [114] proposes a DMS based on a distracted driving decision algorithm which aims to assess and address potential distractions to ensure safe driving practices. It predicts whether the driver is distracted or not.

These papers provide a good overview of the current state of the art in DMS and contribute to the development of advanced DMS technologies, aiming to enhance driver safety, detect distractions, and improve situational awareness on the roads. They employ various techniques, including deep learning, IoT, and machine learning, to create efficient and effective driver monitoring solutions. However, before DMSs can be widely deployed, there are still some challenges that need to be addressed, such as:

- Data collection: It is difficult to collect large datasets of driver behavior representative of the real world, as it is difficult to monitor drivers naturally without disrupting their driving experience.
- Algorithm development: Since the driver behaviors can be subtle and vary from person to person, it is challenging to develop algorithms that can accurately identify driver behaviors in real time.
- Cost: DMS demands the use of specialized sensors and software, making them expensive to implement and maintain.

Additionally, with the development and availability of new sensors, they could be used to improve the accuracy and performance of DMSs; for example, radar sensors could be used to track driver head movements and eye gaze. Besides, autonomous vehicles will not need DMSs in the same way that human-driven vehicles do. However, DMSs could still be used to monitor the state of the driver in autonomous vehicles and to provide feedback to the driver if necessary. Despite these challenges, there is a lot of potential for DMSs to improve road safety and the future of DMSs looks promising. As the technology continues to develop, DMSs could become an essential safety feature in vehicles, both human-driven and autonomous. The list of reviewed papers on driver monitoring system is summarized in Table 5.

### 4.5. Lane Departure Warning System

The Lane Departure Warning System (LDWS) is a type of ADAS that is designed to warn drivers when they are unintentionally drifting out of their lane. LDWSs typically use cameras, radar, lidar, or a combination of sensors to detect the lane markings on the road, and then they use this information to monitor the driver’s position in the lane. If the driver starts to drift out of the lane, the LDWS will sound an audible alert or vibrate the steering wheel to warn the driver. These systems can be a valuable safety feature and are especially helpful for drivers, as they can help to prevent accidents caused by driver drowsiness or distraction and they can help to keep drivers alert and focused on the road.

LDWSs are becoming increasingly common in new vehicles. In fact, according to NHTSA, lane departure crashes account for about 5% of all fatal crashes in the United States and the NHTSA has mandated that all new vehicles sold in the United States be equipped with LDWSs by 2022 [115].

LDWSs can be a valuable safety feature, but they are not perfect. They can sometimes be fooled by objects that look like lane markings, such as shadows or road debris, and may not be accurate when the road markings are faded or obscured. Additionally, LDWS can only warn drivers; they cannot take corrective action on their own, which means they may not be effective for drivers who are drowsy or distracted.

Despite these limitations, LDWS can be a valuable tool for reducing the number of accidents, and are especially beneficial for long-distance driving, as they can help keep drivers alert and focused. They can: (i) help to prevent accidents by alerting drivers to unintentional lane departures, (ii) help drivers stay alert and focused on the road, (iii) be especially helpful for drivers who are drowsy or distracted, (iv) help to keep drivers in their lane, which can improve lane discipline and reduce the risk of sideswipe collisions, thus improving the driver safety and comfort. Therefore, LDWSs are becoming increasingly common in new vehicles, as they greatly reduce drivers’ stress and fatigue.

Overall, LDWSs are a valuable safety feature that can help to prevent accidents, though they are not guaranteed to do so. It is important to remember that these systems are not a substitute for safe driving practices. Drivers should always be alert and focused on the road, aware of their surroundings and use safe driving practices at all times, even when they are using an LDWS.

#### Search Terms and Recent Trends in LDWS

**Figure 6:**
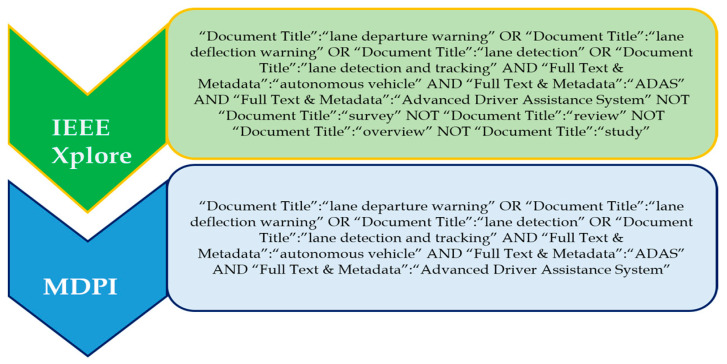
Search queries for each of the databases for the lane departure warning system. The databases include IEEE Xplore and MDPI.

‘Lane departure warning’, ‘lane deflection warning’, ‘lane detection’, and ‘lane detection and tracking’ are four prominent search terms used to investigate the topic. The ‘OR’ operator was used to choose and combine the most relevant and regularly used applicable phrases. The search phrases ‘lane departure warning’, ‘lane deflection warning’, ‘lane detection’, and ‘lane detection and tracking’ were discovered. Figure 6 shows the complete search query for each of the databases. The databases include IEEE Xplore and MDPI.

Lane detection is a critical task in computer vision and autonomous driving systems. These review papers explore various lane detection techniques proposed in recent research papers. The reviewed papers cover diverse approaches, including lightweight CNNs, sequential prediction networks, 3D lane detection, and algorithms for intelligent vehicles in complex environments. The existing lane detection algorithms are not robust to challenging road conditions, such as shadows, rain, and snow, along with occlusion and illumination, and scenarios where lane markings are not visible and are limited in their ability to detect multiple lanes and to accurately estimate the 3D position of the lanes. 

This research review paper examines recent advancements in lane detection techniques, focusing on the integration of DNNs and sensor fusion methodologies. The review encompasses papers published between 2019 and 2022, exploring innovative approaches to improve the robustness, accuracy, and performance of lane detection systems in various challenging scenarios.

The reviewed papers present various innovative approaches for lane detection in the context of autonomous driving systems. Lee et al. [116] introduce a self-attention distillation method to improve the efficiency of lightweight lane detection CNNs without compromising accuracy. FastDraw [117] addresses the long tail of lane detection using a sequential prediction network to consider contextual information for better predictions. 3D-LaneNet [118] incorporates depth information from stereo cameras for end-to-end 3D multiple lane detection. Wang et al. [119] propose a data enhancement technique called Light Conditions Style Transfer for lane detection in low-light conditions, improving model robustness. Other methods explore techniques such as ridge detectors [120], LSTM networks [121], and multitask attention networks [122] to enhance lane detection accuracy in various challenging scenarios. Additionally, some papers integrate multiple sensor data [123,124,125,126] or use specific sensors like radar [127] and light photometry systems [128] to achieve more robust and accurate lane detection for autonomous vehicles. These research contributions provide valuable insights into the development of advanced lane detection systems for safer and more reliable autonomous driving applications.

In their recent research, Lee et al. [116] proposed a novel approach for learning lightweight lane detection CNNs by applying self-attention distillation. FastDraw [117] addressed the long tail of lane detection by using a sequential prediction network to better predict lane markings in challenging conditions. Garnett et al. [118] presented 3D-LaneNet, an end-to-end method incorporating depth information from stereo cameras for 3D multiple lane detection. Additionally, Cao et al. [123] tailored a lane detection algorithm for intelligent vehicles in complex road conditions, enhancing real-world driving reliability. Kuo et al. [129] optimized image sensor processing techniques for lane detection in vehicle lane-keeping systems. Lu et al. [120] improved lane detection accuracy using a ridge detector and regional G-RANSAC. Zou et al. [130] achieved robust lane detection from continuous driving scenes using deep neural networks. Liu et al. [119] introduced Light Conditions Style Transfer for lane detection in low-light conditions. Wang et al. [124] used a map to enhance ego-lane detection in missing feature scenarios. Khan et al. [127] utilized impulse radio ultra-wideband radar and metal lane reflectors for robust lane detection in adverse weather conditions. Yang et al. [121] employed long short-term memory (LSTM) networks for lane position detection. Gao et al. [131] minimized false alarms in lane departure warnings using an Extreme Learning Residual Network and ϵ-greedy LSTM. Moreover, ref. [132] proposed a real-time attention-guided DNN-based lane detection framework and CondLaneNet [133] used conditional convolution for top-to-down lane detection. Dewangan and Sahu [134] analyzed driving behavior using vision-sensor-based lane detection. Haris and Glowacz [135] utilized object feature distillation for lane line detection. Lu et al. [136] combined semantic segmentation and optical flow estimation for fast and robust lane detection. Suder et al. [128] designed low-complexity lane detection methods for light photometry systems. Ko et al. [137] combined key points estimation and point instance segmentation for lane detection. Zheng et al. [138] introduced CLRNet for lane detection, while Wang et al. [122] proposed a multitask attention network (MAN). Khan et al. [139] developed LLDNet, a lightweight lane detection approach for autonomous cars. Chen and Xiang [125] incorporated pre-aligned spatial–temporal attention for lane mark detection. Nie et al. [126] integrated a camera with dual light sensors to improve lane-detection performance in autonomous vehicles. These studies collectively present diverse and effective methodologies, contributing to the advancement of lane-detection systems in autonomous driving and intelligent vehicle applications. The list of reviewed papers on lane-departure warning system is summarized in Table 6.

### 4.6. Forward-Collision Warning System

A Forward-Collision Warning System (FCWS) is a type of ADAS that warns drivers of potential collisions with other vehicles or objects in front of them. FCWSs typically use radar, cameras, or lidar to track the distance and speed of vehicles in front of the vehicle, and they alert the driver if the vehicle is getting too close to the vehicle in front. When the system detects that a collision is imminent, it alerts the driver with a visual or audible warning.

FCWSs can be an invaluable safety feature, as they can help prevent accidents caused by driver distraction or drowsiness. According to the NHTSA, rear-end collisions account for about 25% of all fatal crashes in the United States [140].

FCWSs are becoming increasingly common in new vehicles. The NHTSA has mandated that all new cars sold in the United States come equipped with FCWS systems by 2022.

FCWSs: (i) help prevent accidents caused by driver distraction or drowsiness, (ii) help drivers to brake sooner, which can reduce the severity of rear-end crashes and accidents, (iii) help improve the driver awareness of the surrounding traffic, (iv) help to reduce driver stress and fatigue.

Although FCWSs offer many advantages, they have limitations such as: (i) being less effective in certain conditions, such as heavy rain or snow, (ii) being prone to false alarms, which can lead to driver desensitization, (iii) are not a substitute for safe driving practices, such as paying attention to the road and using turn signals.

Overall, FCWSs can be a valuable safety feature, but they are not guaranteed to prevent accidents. Drivers should still be aware of their surroundings and use safe driving practices at all times.

#### Search Terms and Recent Trends in FCWS

**Figure 7:**
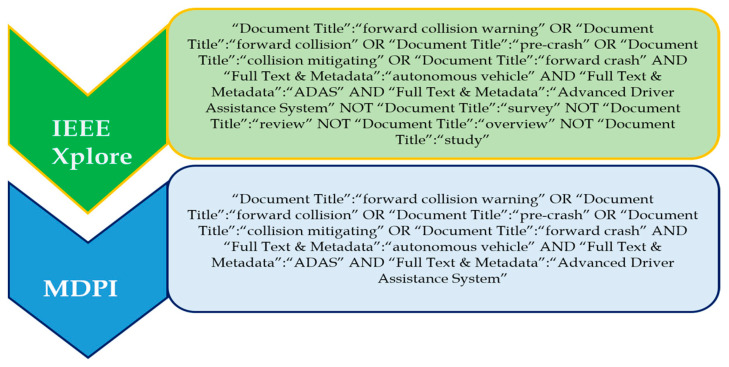
Search queries for each of the databases for the lane-departure warning system. The databases include IEEE Xplore and MDPI.

‘Forward collision warning’, ‘forward collision’, ‘pre-crash’, ‘collision mitigating’, and ‘forward crash’ are the prominent search terms used to investigate this topic. The ‘OR’ operator was used to choose and combine the most relevant and regularly used applicable phrases. That is, the search phrases ‘forward collision warning’, ‘forward collision’, ‘pre-crash’, ‘collision mitigating’, and ‘forward crash’ were discovered. Figure 7 shows the complete search query for each of the databases. The databases include IEEE Xplore and MDPI.

The papers listed discuss the development of FCWSs for autonomous vehicles in recent years. Ref. [141] suggests an autonomous vehicle collision avoidance system that employs predictive occupancy maps to estimate other vehicles’ future positions, enabling collision-free motion planning. Ref. [142] introduces a forward collision prediction system using online visual tracking to anticipate potential collisions based on other vehicles’ positions. Ref. [143] proposes an FCWS that combines driving intention recognition and V2V communication to predict and warn about potential collisions with front vehicles. Ref. [144] presents an FCWS for autonomous vehicles that deploys a CNN to detect and track nearby vehicles. Ref. [145] introduces a real-time FCW technique involving detection and depth estimation networks to identify nearby vehicles and estimate distances. Ref. [146] proposes a vision-based FCWS merging camera and radar data for real-time multi-vehicle detection, addressing challenging conditions like occlusions and lighting variations. Tang et al. [147] introduce a monocular range estimation system using a single camera for precise FCWS, especially in difficult scenarios. Lim et al. [148] suggest a smartphone-based FCWS for motorcyclists utilizing phone sensors to predict collision risks. Farhat et al. [149] present a cooperative FCWS using DL to predict collision likelihood in real time by considering data from both vehicles’ sensors. Hong and Park [150] offer a lightweight FCWS for low-power embedded systems, combining cameras and radar for real-time multi-vehicle detection. Albarella et al. [151] and Lin et al. [152] propose V2X communication-based FCWS, with [151] for electric vehicles and [152] targeting curve scenarios. Yu and Ai [153] suggest a hybrid DL approach employing CNN and recurrent NN for robust FCWS predictions. Olou et al. [154] introduce an efficient CNN model for accurate forward collision prediction, even in challenging conditions. Pak [155] presents a hybrid filtering method that improves radar-based FCWS by fusing data from multiple sensors, enhancing reliability.

This compilation of research papers demonstrates the extensive efforts in the field of forward-collision warning and avoidance systems, which are crucial for enhancing vehicular safety. Lee and Kum [141] propose a ‘Collision Avoidance/Mitigation System’ incorporating predictive occupancy maps for autonomous vehicles. Manghat and El-Sharkawy [142] present ‘Forward Collision Prediction with Online Visual Tracking’, utilizing online visual tracking for collision prediction. Yang, Wan, and Qu [143] introduce ‘A Forward Collision Warning System Using Driving Intention Recognition’, integrating driving intention recognition and V2V communication. Kumar, Shaw, Maitra, and Karmakar [144] offer ‘FCW: A Forward Collision Warning System Using Convolutional Neural Network’, deploying CNN for warning generation. Wang and Lin [145] present ‘A Real-Time Forward Collision Warning Technique’, integrating detection and depth estimation networks for real-time warnings. Lin, Dai, Wu, and Chen [146] introduce a ‘Driver Assistance System with Forward Collision and Overtaking Detection’. Tang and Li [147] propose ‘End-to-End Monocular Range Estimation’ for collision warning. Lim et al. [148] created a ‘Forward Collision Warning System for Motorcyclists’ using smartphone sensors. Farhat, Rhaiem, Faiedh, and Souani [149] present a ‘Cooperative Forward Collision Avoidance System Based on Deep Learning’. Hong and Park [150] propose a ‘Lightweight Collaboration of Detecting and Tracking Algorithm’ for embedded systems. Albarella et al. [151] present a ‘Forward-Collision Warning System for Electric Vehicles’, validated both virtually and in real environments. Liu et al. [152] focus on ‘Forward Collision on a Curve based on V2X’ with a target selection method. Yu and Ai [153] present ‘Vehicle Forward Collision Warning based upon Low-Frequency Video Data’ using hybrid deep learning. Olou, Ezin, Dembele, and Cambier [154] propose ‘FCPNet: A Novel Model to Predict Forward Collision’ based on CNN. Pak [155] contributes ‘Hybrid Interacting Multiple Model Filtering’ to improve radar-based warning reliability. Together, these papers collectively advance the understanding and development of forward collision warning and avoidance systems. The list of reviewed papers on forward-collision warning system is summarized in Table 7.

### 4.7. Blind Spot Detection

Blind spot detection (BSD) is a type of ADAS that helps to prevent accidents by alerting drivers to vehicles, pedestrians, or objects that are in their blind spots. Blind spots are the areas around a vehicle that cannot be seen by the driver when looking in the rear-view or side mirrors. These areas can be especially dangerous when changing lanes, merging onto a highway, or while parking, and it is necessary to present accidents caused by lane changes into the blind spot of other vehicles.

When a vehicle is detected in the blind spot, the system alerts the driver with a visual or audible warning. Some systems will also illuminate a light in the side mirror to indicate that there is a vehicle in the blind spot, while some systems also provide a graphic representation of the vehicle in the blind spot on the dashboard.

BSD systems can be a valuable safety feature and are becoming increasingly common in new vehicles, as they can help to prevent accidents caused by driver inattention or driving changing lanes into other vehicles. They help to reduce the severity of accidents that do occur, thereby reducing drivers’ stress and fatigue and helping drivers to stay alert and more aware of their surroundings. According to the NHTSA, blind spot crashes account for about 2% of all fatal crashes in the United States [57], and the NHTSA has mandated that all new cars sold in the United States come equipped with BSD systems by 2022.

Although BSD has many advantages, it has certain limitations such as: (i) it is less effective in certain conditions, such as heavy rain or snow, (ii) it is prone to false alarms, which can lead to driver desensitization, (iii) it is not a substitute for safe driving practices, such as using turn signals and checking blind spots before changing lanes.

Overall, BSD systems can be a valuable safety feature, but they are not a guarantee against accidents. Drivers should still be aware of their surroundings and use safe driving practices at all times.

#### Search Terms and Recent Trends in Blind Spot Detection

**Figure 8:**
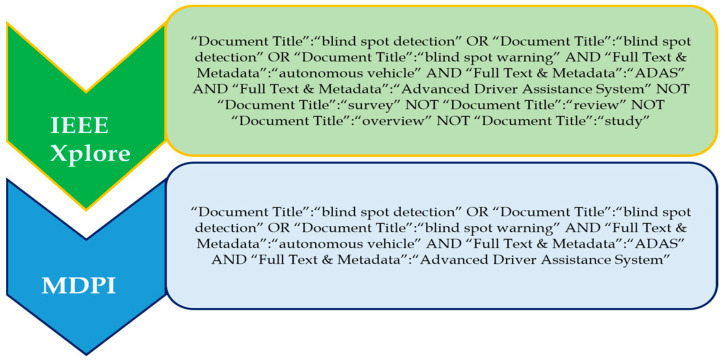
Search queries for each of the databases for blind spot detection. The databases include IEEE Xplore and MDPI.

‘Blind spot’, ‘blind spot detection’, and ‘blind spot warning’, are the three prominent search terms used to investigate this topic. The ‘OR’ operator was used to choose and combine the most relevant and regularly used applicable phrases. That is, the search phrases ‘blind spot’, ‘blind spot detection’, and ‘blind spot warning’, were discovered. Figure 8 shows the complete search query for each of the databases. The databases include IEEE Xplore and MDPI.

The papers mentioned discuss the development of blind-spot detection systems (BSDSs) for vehicles. BSDSs are designed to alert drivers to vehicles that are in their blind spots, where they cannot be seen in their mirrors.

The Gale Bagi et al. [156] paper discusses a BSDS combining radar and cameras for accurate vehicle detection in blind spots. Radar detects vehicles and cameras identify them. Details about sensors and system architecture are necessary for a comprehensive understanding.

Ref. [157] introduces a probabilistic BSDS estimating blind spot risks using vehicle speed, direction, and driver’s blind spot angle. It offers nuanced insights into collision potential, enhancing safe driving.

Zhao et al. [158] propose a promising BSDS using a lightweight NN and cameras for real-time detection. This approach improves detection capabilities with practical design. Chang et al. [159] present an AI-based BSDS warning for motorcyclists using various sensors, proactively detecting blind spot vehicles and enhancing rider safety. Naik et al. [160] propose lidar-based early BSDS, creating a 3D map to detect blind-spot vehicles in advance.

The authors of [161] describe a real-time two-wheeler BSDS using computer vision and ultrasonic sensors, confirming blind spot vehicles. Shete et al. [162] suggest a forklift-specific BSDS using ultrasonic sensors to detect blind spot vehicles and warn drivers. Schlegel et al. [163] propose an optimization-based planner for robots, considering blind spots and other vehicles to ensure safe navigation. Kundid et al. [164] introduce an ADAS algorithm creating a wider view to enhance driver awareness, mitigating blind spot issues.

Sui et al. [165] propose an A-pillar blind spot display algorithm using cameras to show blind spot information on the A-pillar and side mirrors. Wang et al. [166] present a vision-based BSDS using depth cameras to identify blind spot vehicles in a 3D map. Zhou et al. [167] focus on high-speed pedestrians in blind spots, using cameras and radar to detect pedestrians and pre-detection to avoid collisions. Ref. [168] introduces a multi-sensor BSDS for micro e-mobility vehicles, using cameras, radar, ultrasonic sensors, and gesture recognition for better blind-spot awareness. Ref. [169] suggests a multi-deep CNN-based BSDS for commercial vehicles using cameras, effectively addressing blind-spot challenges.

Overall, these papers present a variety of promising methods for developing BSDS. The systems proposed in these papers can detect vehicles in a variety of conditions, and they can be used in a variety of vehicles. The collection of research papers explores a broad spectrum of approaches to address blind spots in various domains, including robotics, automotive applications, and micro e-mobility. The focus ranges from sensor technologies such as cameras, lidar, and ultrasonic sensors to methodologies including AI, probabilistic estimation, and computer vision, introducing innovative algorithms, technologies, and architectures to enhance blind-spot detection, awareness, and collision prevention. The studies emphasize real-time detection, early warning, and proactive risk prediction, all contributing to enhance vehicular safety. The common thread among these studies is their commitment to improving safety by addressing the visibility limitations posed by blind spots. The list of reviewed papers on driver monitoring system is summarized in Table 8.

### 4.8. Emergency Braking System

The Emergency Braking System (EBS), also referred to as automatic emergency braking (AEB), is an ADAS that detects and tracks other vehicles in the vicinity, calculates the risk of a collision, and automatically applies the brakes in the event of an imminent collision to prevent or mitigate a collision. EBS helps to prevent accidents caused by the driver’s inattention, drowsiness, or reaction time. EBSs can be a valuable safety feature, typically using radar, camera, or laser sensors to detect vehicles or objects in front of the car. According to the NHTSA [140], rear-end crashes account for about 25% of all fatal crashes in the United States.

EBSs are becoming increasingly common in new vehicles. In fact, the NHTSA has mandated that all new cars sold in the United States come equipped with EBSs by 2022. EBSs have numerous benefits, as they help to (i) prevent accidents caused by driver distraction or drowsiness, (ii) reduce the severity of accidents that do occur, and (iii) keep drivers alert and focused on the road.

With these benefits comes certain limitations, as these systems are (i) less effective in certain conditions, such as heavy rain or snow, (ii) prone to false alarms, which can lead to driver desensitization, and (iii) not a substitute for safe driving practices, such as paying attention to the road and using turn signals.

Overall, EBSs can be a valuable safety feature, but they are not guaranteed to prevent accidents. Drivers should still be aware of their surroundings and use safe driving practices at all times.

#### Search Terms and Recent Trends in Emergency Braking Systems

**Figure 9:**
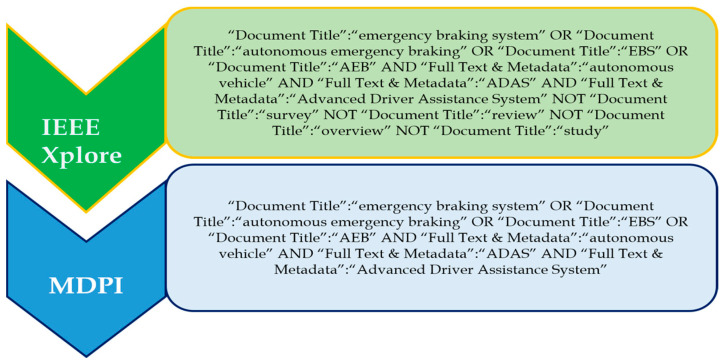
Search queries for each of the databases for the emergency braking system. The databases include IEEE Xplore and MDPI.

‘Emergency braking system’, ‘autonomous emergency braking’, ‘EBS’, and ‘AEB’, are the prominent search terms used to investigate this topic. The ‘OR’ operator was used to choose and combine the most relevant and regularly used applicable phrases. That is, the search phrases ‘emergency braking system’, ‘autonomous emergency braking’, ‘EBS’, and ‘AEB’, were discovered. Figure 9 shows the complete search query for each of the databases. The databases include IEEE Xplore and MDPI.

Flores et al. [170] propose a cooperative car-following and emergency braking system using radar, lidar, and cameras to detect and predict vehicle and pedestrian movements. It automatically applies the brakes to prevent collisions while also facilitating vehicle-to-vehicle communication. Shin et al. [171] introduce an adaptive AEB strategy utilizing radar and cameras to detect and calculate braking forces for front and rear vehicle collision avoidance. It considers speed, distance, and vehicle dynamics for effective collision prevention.

Yang et al. [172] have developed an AEB-P system with radar and cameras, using advanced control to determine braking forces for pedestrian collision avoidance, accounting for pedestrian speed, distance, and vehicle dynamics. Gao et al. [173] present a hardware-in-the-loop simulation platform for AEB system testing across various scenarios, ensuring reliability and effectiveness. Guo et al. [174] introduce a variable time headway AEB algorithm using predictive modeling, combining radar and cameras. It adapts time headway for braking by considering speed, distance, and vehicle dynamics.

Leyrer et al. [175] propose a simulation-based robust AEBS design using optimization techniques to enhance system performance and reliability. Yu et al. [176] introduce an AEBC system utilizing radar and cameras, applying control algorithms to prevent collisions at intersections considering vehicle and pedestrian speed, distance, and dynamics. Izquierdo et al. [177] explore using MEMS microphone arrays for AEBS, improving pedestrian detection through audio cues in a variety of environments.

Jin et al. [178] present an adaptive AEBC strategy for driverless vehicles in campus environments, utilizing radar and cameras to prevent collisions by considering vehicle and pedestrian characteristics and dynamics. Mannam and Rajalakshmi [179] assess AEBS scenarios for autonomous vehicles using radar and cameras, determining collision interventions based on vehicle and pedestrian detection, speed, and distance. Guo et al. [180] study AEBS control for commercial vehicles, considering driving conditions alongside radar and camera-based detection and control algorithms to avoid collisions based on vehicle and pedestrian dynamics.

These papers all represent significant advances in the field of AEB systems. They propose new methods for detecting and tracking vehicles, pedestrians, and environmental features. They also propose new control algorithms for determining the optimal braking force to apply to avoid a collision. These advances have the potential to make AEB systems more effective and reliable and to help prevent traffic accidents.

All the systems discussed were evaluated in a variety of traffic scenarios, and they were shown to be able to significantly reduce the number of accidents. The reviewed papers collectively explore a diverse range of topics within the realm of autonomous emergency braking (AEB) systems for enhanced road safety. 

These topics include cooperative car-following, pedestrian avoidance, collision avoidance with rear vehicles, longitudinal active collision avoidance, hardware-in-the-loop simulation, variable time headway control, environmental feature recognition, simulation-based robust design, inevitable collision state-based control, innovative sensor utilization (MEMS microphone array), adaptive strategies for specific scenarios, determination of AEB-relevant scenarios, and specialized AEB algorithms for commercial vehicles. These contributions highlight the multi-faceted nature of AEB research, highlighting advancements in simulation, sensing, control strategies, and contextual optimization and emphasizing safety, prediction, algorithm optimization, and system validation. As autonomous vehicles continue to evolve, these papers will collectively contribute to enhancing the effectiveness and reliability of AEB systems, thereby advancing road safety in modern transportation and ultimately promoting safer and more reliable autonomous driving experiences. The list of reviewed papers on emergency braking system is summarized in Table 9.

### 4.9. Adaptive Cruise Control

Adaptive cruise control (ACC) is a driver assistance system that automatically adjusts a vehicle’s speed when there are slow-moving vehicles ahead to maintain a safe following distance. When the road ahead is clear, ACC automatically accelerates to the driver’s pre-set speed.

ACC is a Level 1 ADAS feature, which means that it requires some driver input. The driver still needs to be alert and ready to take over if necessary. However, ACC can help to reduce driver fatigue and stress, and it can also help to prevent accidents.

ACC systems typically use a radar sensor to detect the speed and distance of vehicles ahead. The sensor is mounted in the front of the vehicle, and it can typically detect vehicles up to several hundred feet away. The sensor sends this information to a control unit, which then calculates the appropriate speed for the vehicle to maintain a safe following distance. 

ACC systems can be either speed-only or full-range systems. Speed-only systems only adjust the vehicle’s speed, while full-range systems can also brake the vehicle to maintain a safe following distance. Full-range systems are more advanced, and they are typically more expensive. ACC systems can be set to a specific speed, or they can be set to follow the speed of the vehicle ahead. ACC systems can also be set to a maximum following distance, and the system will not allow the vehicle to get closer than the set distance to the vehicle ahead.

ACC systems are becoming increasingly common in vehicles, as they offer several safety and convenience benefits such as reducing traffic congestion and improving fuel efficiency. ACC systems can also help to prevent accidents by reducing the risk of rear-end collisions. They are especially beneficial for long-distance driving, as they can help to reduce driver fatigue. The benefits of ACC systems are as follows:

- Reduced driver fatigue: ACC can help to reduce driver fatigue by taking over the task of maintaining a safe following distance. This can be especially beneficial for long-distance driving.
- Increased safety: ACC can help prevent accidents by automatically adjusting the vehicle’s speed to maintain a safe following distance.
- Improved convenience: ACC can make driving more convenient by allowing the driver to set a cruising speed and then relax.
- Improved fuel efficiency: ACC systems can help to improve fuel efficiency by allowing drivers to maintain a constant speed, which can reduce unnecessary acceleration and braking.

Despite these benefits, ACC systems face numerous challenges, as they are (i) expensive, especially in high-end vehicles, (ii) complex to install and calibrate, which can increase the cost of ownership, and (iii) unreliable in poor weather conditions, such as rain or snow.

Overall, ACC systems are a valuable safety feature that can help to prevent accidents and make driving more convenient. However, they are not without their challenges, such as cost and complexity. As ACC systems become more affordable and reliable, they are likely to become more widespread in vehicles.

#### Search Terms and Recent Trends in Adaptive Cruise Control

**Figure 10:**
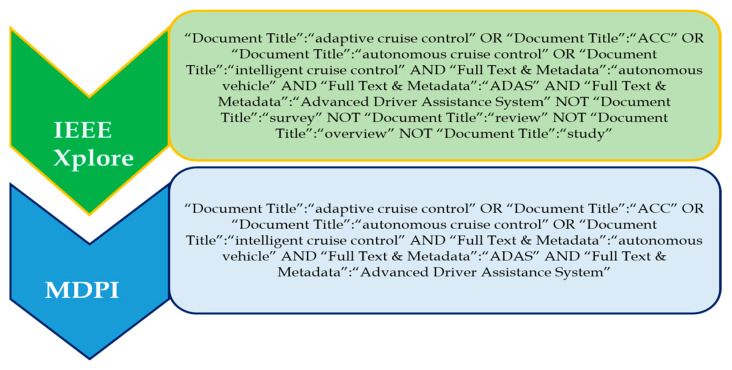
Search queries for each of the databases for the adaptive cruise control system. The databases include IEEE Xplore and MDPI.

‘Adaptive cruise control’, ‘ACC’, ‘autonomous cruise control’, and ‘intelligent cruise control’ are the prominent search terms used to investigate this topic. The ‘OR’ operator was used to choose and combine the most relevant and regularly used applicable phrases. That is, the search phrases ‘adaptive cruise control’, ‘ACC’, ‘autonomous cruise control’, and ‘intelligent cruise control’ were discovered. Figure 10 shows the complete search query for each of the databases. The databases include IEEE Xplore and MDPI.

G. Li and D. Görges [181] propose an innovative approach combining ecological ACC and energy management for HEVs using heuristic dynamic programming. The algorithm optimizes speed profiles, considering traffic conditions, state of charge, and driver preferences for fuel efficiency and comfort. S. Cheng et al. [182] discuss a multiple-objective ACC with dynamic velocity obstacle (DYC) prediction, optimizing speed, acceleration, safety, comfort, and fuel efficiency by forecasting surrounding vehicle trajectories. J. Lunze [183] introduces an ACC strategy ensuring collision avoidance through predictive control using a combination of predictive control and MPC to optimize vehicle speed profiles. Woo, H. et al. [184] enhance ACC safety and efficiency through operation characteristic estimation and trajectory prediction. Their work adjusts speed and acceleration considering vehicles’ dynamics and surroundings.

Zhang, S. and Zhuan, X. [185] developed an ACC for BEVs that accounts for weight changes. Weight adjustments based on battery discharge and passenger load are used to ensure safe and comfortable driving. C. Zhai et al. [186] present an ecological CAC strategy for HDVs with time delays using distributed algorithms for platoon coordination, achieving fuel efficiency and ecological benefits. Li and Görges [187] designed an ecological ACC for step-gear transmissions using reinforcement learning. It optimizes fuel efficiency while maintaining safety through learned intelligent control strategies. Jia, Jibrin, and Görges [188] propose an energy-optimal ACC for EVs using linear and nonlinear MPC techniques, minimizing energy consumption based on dynamic driving and traffic conditions. Nie and Farzaneh [189] focus on eco-driving ACC with an MPC algorithm for reduced fuel consumption and emissions while ensuring safety and comfort. Guo, Ge, Sun, and Qiao [190] introduce an MPC-based ACC with relaxed constraints to enhance fuel efficiency while considering speed limits and safety distances for driving comfort.

Liu, Wang, Hua, and Wang [191] analyze CACC safety with communication delays using MPC and fuzzy logic to ensure stable and effective CACC operation under real-world communication conditions. Lin et al. [192] compare DRL and MPC for ACC, suggesting a hybrid approach for improved fuel efficiency, comfort, and stability. Gunter et al. [193] investigate the string stability of commercial ACC systems, highlighting potential collision risks in platooning situations and recommending improvements. Sawant et al. [194] present a robust CACC control algorithm using MPC and fuzzy logic to ensure safe operation even with limited data on preceding vehicle acceleration. Yang, Wang, and Yan [195] optimize ACC through a combination of MPC and ADRC, enhancing fuel efficiency and robustness to disturbances. Anselma [196] proposes a powertrain-oriented ACC considering fuel efficiency and passenger comfort using MPC and powertrain modeling.

Chen [197] designed an ACC tailored to cut-in scenarios using MPC for fuel efficiency optimization during lane changes. Hu and Wang [198] introduce a trust-based ACC with individualization using a CBF approach, allowing vehicles to have personalized safety requirements. Yan et al. [199] hybridized DDPG and CACC for optimized traffic flow, leveraging learning-based and cooperative techniques. Zhang et al. [200] created a human-lead-platooning CACC to integrate human-driven vehicles into platoons. The author of [201] presents a resilient CACC using ML to enhance robustness and adaptability to uncertainties and disruptions. Kamal et al. [202] propose an ACC with look-ahead anticipation for freeway driving, adjusting control inputs based on predicted traffic conditions. Li et al. [203] leverage variable compass operator pigeon-inspired optimization (VCPO-PIO) for ACC control input optimization. Petri et al. [204] address ACC for EVs with FOC, considering unique characteristics and energy management needs. The list of reviewed papers on adaptive cruise control is summarized in Table 10.

### 4.10. Around-View Monitoring (AVM)

Around-View Monitoring (AVM) is an ADAS that uses multiple cameras to provide a 360-degree view of the vehicle’s surroundings. This helps drivers to see more of what is around them, which can improve safety and make it easier to park. It is especially helpful in tight spaces or when backing up.

AVM systems typically use four cameras, one mounted on each side of the vehicle and one in the rear. The cameras are connected to a central computer, which stitches the images together to create a panoramic view of the vehicle’s surroundings. This view is displayed on a screen in the vehicle’s cabin, giving the driver a bird’s-eye view of what is around them and preventing blind spots. Thus, AVM systems are a valuable safety feature and can be used for a variety of purposes, including parking, backing up, maneuvering in tight spaces, monitoring blind spots, and overall enhancing safety by giving drivers a better view of their surroundings and preventing accidents, especially in low-visibility conditions. The challenges of AVM in ADAS are their high cost and complexity of installation.

The ADAS features with which AVM are often combined include blind-spot detection, lane departure warning system, a forward collision warning system, and parking assistance systems. Overall, these features can work together to provide drivers with a more comprehensive view of their surroundings, help them avoid accidents, and make it easier to park.

#### Search Terms and Recent Trends in Around-View Monitoring

**Figure 11:**
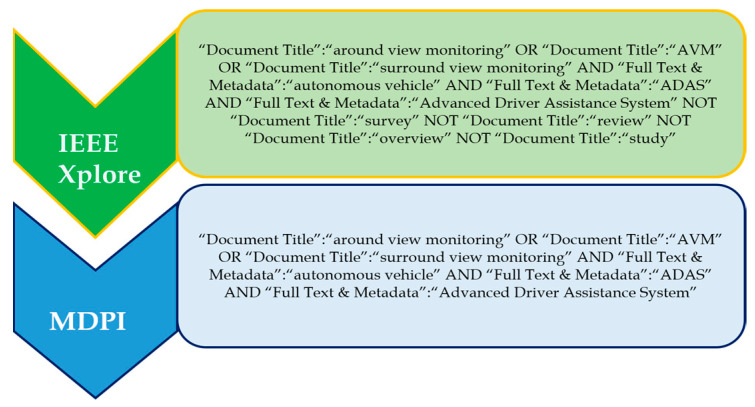
Search queries for each of the databases for around view monitoring. The databases include IEEE Xplore and MDPI.

‘Around view monitoring’, ‘AVM’, and ‘surround view monitoring’ are the prominent search terms used to investigate this topic. The ‘OR’ operator was used to choose and combine the most relevant and regularly used applicable phrases. That is the search phrases ‘around view monitoring’, ‘AVM’, and ‘surround view monitoring’ were discovered. Figure 11 shows the complete search query for each of the databases. The databases include IEEE Xplore and MDPI.

Ref. [205] introduces a novel method by integrating semantic segmentation with AVM for lane-level localization. Utilizing visual data and semantic information, a DL model segments lanes and localizes the vehicle, enhancing navigation precision and safety. Refs. [206,207] integrate motion estimation into an AVM for ADAS. The author of [206] employs a Kalman filter to estimate motion, improving AVM image accuracy by up to 20%. The author of [207] focuses on homogeneous surfaces, achieving 90% accuracy with image registration and optical flow. Ref. [208] discusses AVM/lidar sensor fusion for parking-based SLAM. The fusion creates a map for SLAM and parking detection, with an improved loop closure accuracy of 95%. 

Ref. [209] proposes AVM-based parking space detection using image processing and machine learning, providing an effective solution. Ref. [210] presents automatic AVM camera calibration using image processing and machine learning, streamlining the process without a physical calibration rig. Ref. [211] enhances AVM image quality via synthetic image learning for deblurring, addressing blurriness and distortion. Ref. [212] introduces AVM calibration using unaligned square boards, simplifying the process and increasing accuracy without a physical rig. Ref. [213] proposes an AVM-based automatic parking system using parking line detection, offering an accurate and efficient solution. Ref. [214] suggests a DL-based approach to detect parking and collision risk areas in autonomous parking scenarios, improving accuracy and collision assessment.

The papers discussed above provide a good overview of the current state-of-the-art approaches using AVM systems for lane-level localization, motion estimation, parking space detection, and collision risk area detection and improving the performance of AVM systems. The methods proposed in these papers have the potential to significantly improve the safety and efficiency of AVM systems, which in turn improves driving and parking efficiencies, and they are likely to become increasingly common in the future.

These amalgamations of these research papers collectively introduce innovative approaches ranging from semantic segmentation for lane-level localization to motion estimation techniques for enhancing monitoring accuracy, and collectively focus on crucial aspects such as automatic calibration, image-quality enhancement, parking-line detection, and collision-risk assessment. Additionally, by employing advanced techniques like supervised deblurring and DL, the integration of sensor fusion, such as AVM and lidar, significantly improves AVM systems’ reliability, accuracy, and safety, offering promising outcomes for applications like autonomous parking. The synthesis of these diverse techniques showcases the recent advancements and growing potential of AVM in improving vehicle navigation, parking, and overall safety, thus revolutionizing vehicle navigation, parking, and overall driving experiences. The list of reviewed papers on around view monitoring is summarized in Table 11.

## 5. Discussion Datasets

The input data are the most important factor for the ADAS functionalities discussed in this paper. The preparation of the dataset is essential for the DL approaches, particularly in the training phase. The quality of the dataset preparation in the network model determines how well the autonomous car can manage its behavior and make decisions.

A review of journal articles, conference papers, and book chapters found that many studies used self-collected data or collected data online. Some researchers compiled their own dataset for training and then compared it to a publicly available benchmark dataset. Others only used self-collected data for training and validation. Still, others relied only on publicly available datasets for training and validation.

The choice of dataset preparation method depends on the specific research and the availability of resources. Self-collected data can be more representative of the specific environment in which the autonomous car will be operating, but it can be more time-consuming and expensive to collect. Publicly available datasets are more convenient to use, but they may not be as representative of the specific environment. Table 12 lists various public datasets used for different state-of-the-art methods discussed in Section 4.1, Section 4.2, Section 4.3, Section 4.4, Section 4.5, Section 4.6, Section 4.7, Section 4.8, Section 4.9 and Section 4.10.

Besides employing publicly available, free-to-use open-source datasets, the most recent state-of-the-art work uses a self-collected dataset and proposes datasets suitable for their proposed works and makes their proposed dataset available for other researchers. For instance, ref. [40] manually constructed a dataset containing 316 vehicle clusters and 224 non-vehicle clusters, ref. [47] used datasets generated from the transformed results that demonstrate significant improvement, and ref. [62] initially generated a template of a pedestrian from a training dataset. The template was then used to match pedestrians in the lidar point cloud. The authors of the paper evaluated their method based on a dataset of lidar point clouds. Additionally, ref. [63] was evaluated using their dataset and [67] was evaluated using a dataset of images captured in hazy weather, ref. [66] was trained and tested on a dataset of images captured in different weather conditions, ref. [67] was trained on a dataset of images from rural roads, ref. [68] was trained on infrared images captured during nighttime, and ref. [69] was trained on a dataset of images collected from different scenarios, including urban roads, highways, and intersections. If a public dataset is unavailable and the target is specific to a country, as was the case for [91], in which a public dataset for Taiwan was not available, the author evaluated their method based on a locally built dataset [248]. On the other hand, many publications do not mention exactly which dataset was used, instead highlighting that ‘the proposed method was evaluated on a publicly available dataset’ [94,95,96].

In addition to the state-of-the-art methods discussed in the above sections, some of the other notable publications are:

The paper [249] provides a comprehensive overview of the advancements and techniques in object detection facilitated by DL methodologies. The authors survey the state-of-the-art approaches up to the time of publication in 2019, and discuss various DL architectures and algorithms used for object detection, including two-stage detectors, one-stage detectors, anchor-based and anchor-free methods, RetinaNet, and FPNs, along with methodologies handling small objects, occlusions, and cluttered backgrounds. Additionally, they present some promising research directions for future work, such as multi-task learning, attention mechanisms, weakly supervised learning, and domain adaptation. Additionally, their paper explores the architectural evolution of DL models for object detection, discussing the transition from traditional methods to the emergence of region-based and anchor-based detectors, as well as the introduction of feature pyramid networks. The review also covers commonly used datasets for object detection, highlighting their significance in benchmarking algorithms, and discusses the evaluation metrics used to assess the performance of object detection models.

The paper [250] serves as a thorough survey of driving monitoring and assistance systems (DMAS), covering a wide range of technologies and methodologies such as driver monitoring systems (DMS), advanced driver assistance systems (ADAS), autonomous emergency braking (AEB), lane-departure warning systems (LDWS), adaptive cruise control (ACC), and blind spot monitoring (BSM). It explores various aspects of systems designed to monitor driver behavior and provide assistance, contributing to the understanding of advancements in the field of intelligent transportation systems. The comprehensive nature of the survey suggests an in-depth examination of existing technologies, challenges, and potential future directions for driving monitoring and assistance systems.

The paper [251] proposes a novel approach to 3D object detection utilizing monocular images. The key focus is on the use of a Proposal Generation Network tailored for 3D object detection, which integrates depth information derived from monocular images to generate proposals efficiently, contributing to improve the overall accuracy and efficiency of 3D object detection. The paper addresses the challenge of 3D object detection using only monocular images, which is a significant contribution, as many real-world applications rely on single-camera setups.

The paper [252] presents an innovative one-stage approach to monocular 3D object detection, streamlining the detection pipeline and potentially improving real-time performance compared to traditional two-stage approaches, emphasizing the use of discrete depth and orientation representations that suggest a departure from continuous representations, potentially leading to more interpretable and efficient models of the detection process.

The paper [253] explores the integration of AI techniques for object detection and distance measurement in which the algorithms are employed to identify and locate objects in images or videos. Once the objects have been detected, the model estimates their distance from the camera using various techniques, such as depth estimation networks, monocular depth estimation, and stereo depth estimation. This AI-based approach to object detection and distance measurement has the potential to revolutionize various fields. It offers high accuracy, real-time performance, and low cost, making it a promising solution for a wide range of applications.

## 6. Conclusions and Future Trends

Various ADASs discussed in the previous section have the potential to revolutionize the way we drive. By improving road safety, reducing driver workload, and providing a more comfortable and enjoyable driving experience, ADASs can make our roads safer and our journeys more enjoyable.

These DL algorithms are still under development, but they have the potential to revolutionize the way ADASs are designed and implemented. As these algorithms become more powerful and efficient, they will become more widely used in ADASs. Some of the advantages of using deep learning for object detection, recognition, and tracking in ADAS are as follows:

- Accuracy: Deep learning algorithms have been shown to be more accurate than traditional algorithms, especially in challenging conditions.
- Speed: Deep learning algorithms can be very fast, which is important for real-time applications.
- Scalability: Deep learning algorithms can be scaled to handle large datasets and complex tasks.
- Robustness: Deep learning algorithms are relatively robust to noise and other disturbances.

These advantages come with some of the challenges of using DL for object detection, recognition, and tracking in ADAS:

- Data requirements: Deep learning algorithms require large datasets of labeled data to train. This can be a challenge to obtain, especially for rare or unusual objects.
- Computational requirements: Deep learning algorithms can be computationally expensive, which can limit their use in real-time applications.
- Interpretability: Deep learning algorithms are often difficult to interpret, which can make it difficult to understand why they make certain decisions.

Researchers are working on developing newer algorithms and improvising the existing algorithms and techniques to address these challenges. As a result, ADASs are becoming increasingly capable of detecting and tracking objects in a variety of challenging conditions.

ADASs are still under development, but they have the potential to revolutionize the way we drive. By making our roads safer and more efficient, ADASs can help to create a better future for transportation.

ADASs are not without their drawbacks. They can be expensive, and they can sometimes malfunction. Additionally, drivers may become too reliant on ADASs and become less attentive to their driving.

Overall, ADASs offer numerous potential benefits for safety and convenience. However, it is important to be aware of the drawbacks and to use these systems responsibly.

The ongoing continuous advancements and researches are focusing on overcoming the existing drawbacks and the same can be foreseen as the future trends of ADAS.

- Multi-sensor fusion: ADASs are increasingly using multiple sensors, such as cameras, radar, and lidar, to improve the accuracy and reliability of object detection. Multi-sensor fusion can help to overcome the limitations of individual sensors, such as occlusion and poor weather conditions.
- Deep learning: DL is rapidly becoming the dominant approach for object detection, recognition, and tracking in ADAS. Deep learning algorithms are very effective at learning the features that are important for identifying different objects.
- Real-time performance: ADASs must be able to detect, recognize, and track objects in real time. This is essential for safety-critical applications, as delays in detection or tracking can lead to accidents.
- Robustness to challenging conditions: ADASs must be able to operate in a variety of challenging conditions, such as different lighting conditions, weather conditions, and road conditions. Researchers are working on developing new algorithms and techniques to improve the robustness of ADASs to challenging conditions.
- Integration with other ADAS features: ADASs are seeing increased integration with other ADAS features, such as collision avoidance, lane departure warning, and adaptive cruise control. This integration can help to improve the overall safety of vehicles.

These are just some of the future trends in object detection, recognition, and tracking for ADAS. As research in this area continues, ADASs are becoming increasingly capable of detecting and tracking objects in a variety of challenging conditions. This will help to make vehicles safer and more reliable.

Some additional trends that are worth mentioning could be:

- The use of synthetic data: Synthetic data are being used increasingly often to train object detection, recognition, and tracking algorithms. Synthetic data are generated by computer simulations, and they can be used to create training datasets that are more diverse and challenging than the real-world datasets. This might enhance the efficiency of the neural networks, as they can be trained with a combination of real-world datasets supplemented with the synthetic datasets.
- The use of edge computing: Edge computing is a distributed computing paradigm that brings computation and storage closer to the edge of the network. Edge computing can be used to improve the performance and efficiency of ADASs by performing object detection, recognition, and local tracking on the vehicle, implying that the greater the storage on the ADAS implement vehicles, the better the performance of the ADASs.
- The use of 5G: 5G is the next generation of cellular network technology. 5G will offer much higher bandwidth and lower latency than 4G, which will make it possible to stream high-definition video from cameras to cloud-based servers for object detection, recognition, and tracking. Thus, a better cellular network will aid in the continuous training of the NNs and greatly improve the performance with newer data from real environments.

These are just some of the future trends that are likely to shape the development of object detection, recognition, and tracking for ADAS in the years to come.

## Figures and Tables

**Table 1:** Summary of the advantages and disadvantages of each sensor and combinations used in ADAS applications.

Sensors	Advantages	Disadvantages
**Camera**	Relatively inexpensive; Easy to use; High-resolution images.	Affected by environmental factors (lighting, weather); Difficult to interpret images in low-visibility conditions; Can be fooled by glare and reflections; Can only detect objects in the visible spectrum.
**Radar**	Can detect objects at a longer range than cameras, even in poor visibility; Less affected by weather conditions; Can be used to estimate the speed of objects.	Lower resolution than cameras; More expensive than cameras; Can be complex to integrate into vehicles.
**Lidar**	Not affected by environmental factors; Accurate measurement of distance, speed, and shape of objects.	Expensive; Difficult to mount on vehicles; Can produce sparse point clouds; Can be limited in field of view (FOV).
**Camera–Radar Fusion**	Combines the strengths of cameras and lidar sensors; Can be used in challenging weather conditions.	More expensive than using a single sensor; Can be complex to implement.
**Camera–Lidar Fusion**	Combines the strengths of cameras and lidar; Can provide accurate 3D measurements of objects; Robust object detection and tracking system; Can be used in challenging weather conditions.	More expensive than a camera or lidar alone; Can be computationally complex.
**Radar–Lidar Fusion**	Combines the strengths of radar and lidar sensors; Improves accuracy of object detection and tracking in challenging weather conditions.	More expensive than a camera or lidar alone; Can be computationally complex.
**Lidar–Lidar Fusion**	Combines data from multiple lidar sensors; Can improve the accuracy of 3D mapping and object detection; More accurate and reliable object detection and tracking system.	More expensive than lidars alone; Can be computationally complex.

**Table 2:** Chosen publications regarding vehicle detection, their source title, and their number of citations.

SI No.	Ref.	Year	Source Title	Citations
1	[33]	2019	IEEE Transactions on Intelligent Transportation Systems	165
2	[34]	2019	IEEE/CVF International Conference on Computer Vision (ICCV)	88
3	[35]	2019	IEEE International Conference on Robotics and Automation (ICRA)	79
4	[36]	2019	MDPI Intelligent Sensors	58
5	[37]	2019	MDPI Intelligent Sensors	42
6	[38]	2019	MDPI Remote Sensors	41
7	[39]	2020	IEEE Transactions on Vehicular Technology	47
8	[40]	2020	IEEE Journal of Selected Topics in Applied Earth Observations and Remote Sensing	44
9	[41]	2020	IEEE Access	38
10	[42]	2020	MDPI Sensors	56
11	[43]	2020	MDPI Sensors	27
12	[44]	2020	MDPI Remote Sensing	27
13	[45]	2021	IEEE Transactions on Intelligent Transportation Systems	52
14	[46]	2021	IEEE Transactions on Intelligent Transportation Systems	48
15	[47]	2021	IEEE Transactions on Intelligent Transportation Systems	47
16	[48]	2021	MDPI Remote Sensing	37
17	[49]	2021	MDPI Remote Sensing	30
18	[50]	2021	MDPI Sensors	11
19	[51]	2022	IEEE Transactions on Circuits and Systems for Video Technology	20
20	[52]	2022	IEEE Access	13
21	[53]	2022	IEEE Transactions on Intelligent Transportation Systems	12
22	[54]	2022	MDPI Electronics	21
23	[55]	2022	MDPI Sensors	10
24	[56]	2022	MDPI Electronics	6

**Table 3:** Chosen publications regarding pedestrian detection, their source title, and their number of citations.

SI No.	Ref.	Year	Source Title	Citations
1	[58]	2019	IEEE/CVF Conference on Computer Vision and Pattern Recognition (CVPR)	186
2	[59]	2019	IEEE/CVF Conference on Computer Vision and Pattern Recognition (CVPR)	163
3	[60]	2019	2019 IEEE/CVF International Conference on Computer Vision (ICCV)	111
4	[61]	2019	MDPI Sensors	45
5	[62]	2019	MDPI Electronics	26
6	[63]	2019	MDPI Applied Sciences	15
7	[64]	2020	IEEE Transactions on Industrial Electronics	98
8	[65]	2020	2020 IEEE/CVF Conference on Computer Vision and Pattern Recognition (CVPR)	76
9	[66]	2020	IEEE Transactions on Image Processing	42
10	[67]	2020	MDPI Electronics	49
11	[68]	2020	MDPI Applied Science	28
12	[69]	2020	MDPI Sensors	14
13	[70]	2021	IEEE Transactions on Image Processing	54
14	[71]	2021	IEEE/CVF Conference on Computer Vision and Pattern Recognition (CVPR)	45
15	[72]	2021	IEEE Transactions on Intelligent Transportation Systems	27
16	[73]	2021	MDPI Sensors	21
17	[74]	2021	MDPI Sensors	19
18	[75]	2021	MDPI Electronics	15
19	[76]	2022	IEEE Transactions on Circuits and Systems for Video Technology	15
20	[77]	2022	IEEE Transactions on Intelligent Transportation Systems	12
21	[78]	2022	IEEE Transactions on Intelligent Transportation Systems	10
22	[79]	2022	MDPI Sensors	20
23	[80]	2022	MDPI Applied Sciences	11
24	[81]	2022	MDPI Sensors	11

**Table 4:** Chosen publications, source title, and the number of citations for traffic signs detection.

SI No.	Ref.	Year	Source Title	Citations
1	[82]	2019	IEEE Transactions on Image Processing	118
2	[83]	2019	IEEE Transactions on Intelligent Transportation Systems	96
3	[84]	2019	IEEE Access	53
4	[85]	2019	IEEE Transactions on Intelligent Transportation Systems	50
5	[86]	2019	MDPI Sensors	66
6	[87]	2019	MDPI Sensors	44
7	[88]	2020	IEEE Access	151
8	[89]	2020	IEEE Transactions on Intelligent Transportation Systems	131
9	[90]	2020	IEEE Transactions on Intelligent Transportation Systems	52
10	[91]	2020	MDPI Applied Sciences	46
11	[92]	2020	MDPI Electronics	38
12	[93]	2020	MDPI Sensors	16
13	[94]	2021	IEEE Access	63
14	[95]	2021	IEEE Access	30
15	[96]	2021	IEEE Access	19
16	[97]	2021	IEEE Access	16
17	[98]	2021	MDPI Sensors	25
18	[99]	2020	MDPI Sensors	3
19	[100]	2022	IEEE Transactions on Intelligent Transportation Systems	15
20	[101]	2022	IEEE Transactions on Vehicular Technology	11
21	[102]	2022	IEEE Transactions on Intelligent Transportation Systems	11
22	[103]	2022	MDPI Entropy	13
23	[104]	2022	MDPI Symmetry	8
24	[105]	2022	MDPI Sensors	7

**Table 5:** Chosen publications, source title, and the number of citations referring to the driver monitoring system.

SI No.	Ref.	Year	Source Title	Citations
1	[106]	2019	IEEE International Symposium on Robotic and Sensors Environments	2
2	[107]	2019	International Conference on Robot and Human Interactive Communication	1
3	[108]	2019	MDPI Sensors	28
4	[109]	2020	International Conference on Artificial Intelligence in Information and Communication	2
5	[110]	2020	6th International Conference on Interactive Digital Media	1
6	[111]	2021	2nd International Conference on Communication, Computing and Industry 4.0	1
7	[112]	2021	IEEE International Conference on Consumer Electronics and Computer Engineering	-
8	[113]	2022	Interdisciplinary Research in Technology and Management	-
9	[114]	2022	13th International Conference on Information and Communication Technology Convergence	-

**Table 6:** Chosen publications, source title, and the number of citations related to a lane-departure warning system.

SI No.	Ref.	Year	Source Title	Cited by
1	[116]	2019	IEEE/CVF International Conference on Computer Vision	253
2	[117]	2019	IEEE/CVF Conference on Computer Vision and Pattern Recognition	78
3	[118]	2019	IEEE/CVF International Conference on Computer Vision	57
4	[123]	2019	MDPI Sensors	34
5	[129]	2019	MDPI Sensors	16
6	[120]	2019	MDPI Sensors	12
7	[130]	2020	IEEE Transactions on Vehicular Technology	165
8	[119]	2020	IEEE Intelligent Vehicles Symposium (IV)	32
9	[124]	2020	IEEE Access	9
10	[127]	2020	MDPI Sensors	14
11	[121]	2020	MDPI Sensors	9
12	[131]	2020	MDPI Sensors	6
13	[132]	2021	IEEE/CVF Conference on Computer Vision and Pattern Recognition	60
14	[133]	2021	IEEE/CVF International Conference on Computer Vision	44
15	[134]	2021	IEEE Sensors Journal	40
16	[135]	2021	MDPI Electronics	17
17	[136]	2021	MDPI Sensors	14
18	[128]	2021	MDPI Electronics	12
19	[137]	2022	IEEE Transactions on Intelligent Transportation Systems	54
20	[138]	2022	IEEE/CVF Conference on Computer Vision and Pattern Recognition	17
21	[122]	2022	IEEE Transactions on Neural Networks and Learning Systems	15
22	[139]	2022	MDPI Sensors	4
23	[125]	2022	MDPI Sensors	2
24	[126]	2022	MDPI Electronics	-

**Table 7:** Chosen publications, source title, and the number of citations related to forward-collision warning systems.

SI No.	Ref.	Year	Source Title	Cited by
1	[141]	2019	IEEE Access	48
2	[142]	2019	IEEE International Conference on Vehicular Electronics and Safety (ICVES)	2
3	[143]	2020	IEEE Access	31
4	[144]	2020	IEEE International Conference on Electrical and Electronics Engineering (ICE3)	2
5	[145]	2020	IEEE International Conference on Systems, Man, and Cybernetics (SMC)	-
6	[146]	2020	MDPI Sensors	26
7	[147]	2020	MDPI Sensors	4
8	[148]	2021	IEEE Journal of Intelligent and Connected Vehicles	1
9	[149]	2021	IEEE International Conference on Developments in eSystems Engineering (DeSE)	-
10	[150]	2021	IEEE Twelfth International Conference on Ubiquitous and Future Networks (ICUFN)	-
11	[151]	2021	MDPI Energies	-
12	[152]	2022	7th International Conference on Intelligent Informatics and Biomedical Science (ICIIBMS)	1
13	[153]	2022	IEEE 25th International Conference on Intelligent Transportation Systems (ITSC)	-
14	[154]	2022	22nd International Conference on Control, Automation and Systems (ICCAS)	-
15	[155]	2022	MDPI Sensors	3

**Table 8:** Chosen publications, source title, and the number of citations related to driver monitoring systems.

SI No.	Ref.	Year	Source Title	Number of Citations
1	[156]	2019	2019 International Conference on Control, Automation and Information Sciences (ICCAIS)	3
2	[157]	2019	IEEE Intelligent Transportation Systems Conference (ITSC)	1
3	[158]	2019	MDPI Electronics	16
4	[159]	2020	International Symposium on Computer, Consumer, and Control (IS3C)	1
5	[160]	2020	International Conference on Smart Electronics and Communication (ICOSEC)	-
6	[161]	2021	5th International Conference on Electronics, Communication and Aerospace Technology (ICECA)	1
7	[162]	2021	IEEE International Conference on Technology, Research, and Innovation for Betterment of Society (TRIBES)	-
8	[163]	2021	European Conference on Mobile Robots (ECMR)	-
9	[164]	2021	Zooming Innovation in Consumer Technologies Conference (ZINC)	-
10	[165]	2022	IEEE 5th International Conference on Computer and Communication Engineering Technology (CCET)	-
11	[166]	2022	IEEE Intl Conf on Dependable, Autonomic and Secure Computing, Intl Conf on Pervasive Intelligence and Computing, Intl Conf on Cloud and Big Data Computing, Intl Conf on Cyber Science and Technology Congress (DASC/PiCom/CBDCom/CyberSciTech)	-
12	[167]	2022	IEEE 25th International Conference on Intelligent Transportation Systems (ITSC)	-
13	[168]	2022	MDPI Sensors	2
14	[169]	2022	MDPI Sensors	1

**Table 9:** Chosen publications, source title, and the number of citations related to the emergency braking system.

SI No.	Ref.	Year	Source Title	Cited by
1	[170]	2019	IEEE Transactions on Intelligent Transportation Systems	31
2	[171]	2019	IEEE Intelligent Transportation Systems Conference (ITSC)	5
3	[172]	2019	MDPI Sensors	43
4	[173]	2019	IEEE 23rd International Conference on Intelligent Transportation Systems (ITSC)	4
5	[174]	2019	Chinese Automation Congress (CAC)	4
6	[175]	2019	IEEE Intelligent Vehicles Symposium (IV)	-
7	[176]	2020	American Control Conference (ACC)	2
8	[177]	2020	MDPI Sensors	2
9	[178]	2020	International Conference on Advanced Mechatronic Systems (ICAMechS)	-
10	[179]	2020	IEEE Global Conference on Computing, Power, and Communication Technologies (GlobConPT)	-
11	[180]	2020	MDPI Machines	4

**Table 10:** Chosen publications, source title, and the number of citations related to adaptive cruise control.

SI No.	Ref.	Year	Source Title	Number of Citations
1	[181]	2019	IEEE Transactions on Intelligent Transportation Systems	57
2	[182]	2019	IEEE Transactions on Vehicular Technology	54
3	[183]	2019	IEEE Transactions on Intelligent Transportation Systems	39
4	[184]	2019	MDPI Applied Sciences	9
5	[185]	2019	MDPI Symmetry	9
6	[186]	2020	IEEE Access	39
7	[187]	2020	IEEE Transactions on Intelligent Transportation Systems	29
8	[188]	2020	IEEE Transactions on Vehicular Technology	25
9	[189]	2020	MDPI Applied Sciences	29
10	[190]	2020	MDPI Applied Sciences	12
11	[191]	2020	MDPI Sustainability	11
12	[192]	2021	IEEE Transactions on Intelligent Vehicles	69
13	[193]	2021	IEEE Transactions on Intelligent Transportation Systems	68
14	[194]	2021	IEEE Transactions on Intelligent Transportation Systems	31
15	[195]	2021	MDPI Actuators	16
16	[196]	2021	MDPI Energies	13
17	[197]	2021	MDPI Applied Sciences	12
18	[198]	2022	IEEE Transactions on Intelligent Transportation Systems	12
19	[199]	2022	IEEE Transactions on Automation Science and Engineering	10
20	[200]	2022	IEEE Transactions on Intelligent Transportation Systems	8
21	[201]	2022	IEEE Transactions on Intelligent Transportation Systems	8
22	[202]	2022	MDPI Applied Sciences	5
23	[203]	2022	MDPI Electronics	1
24	[204]	2022	MDPI Applied Sciences	1

**Table 11:** Chosen publications, source title, and the number of citations related to around-view monitoring.

SI No.	Ref.	Year	Source Title	Cited by
1	[205]	2019	IEEE Sensors Journal	18
2	[206]	2019	7th International Conference on Mechatronics Engineering (ICOM)	-
3	[207]	2019	7th International Conference on Mechatronics Engineering (ICOM)	-
4	[208]	2019	MDPI Sensors	10
5	[209]	2019	MDPI Applied Sciences	9
6	[210]	2020	IEEE Access	3
7	[211]	2021	17th International Conference on Machine Vision and Applications (MVA)	1
8	[212]	2021	MDPI Sensors	2
9	[213]	2021	MDPI Applied Sciences	1
10	[214]	2022	MDPI Sensors	1

**Table 12:** Datasets employed by the references chosen in this review paper.

SI No.	Name.	Categories	No. of Objects	Papers Used
1	KITTI Vision Benchmark Suite [215,216]	Vehicles, pedestrians, cyclists, and road objects	Over 70,000 images & 30,000 Lidar scans	[33,34,35,37,41,43,46,50,58,59,65,77,78,118,121,124,126,129,133,141,145,151,154,157,170,208]
2	Argoverse [217]	Vehicles, pedestrians, cyclists, traffic lights, road objects, and more	Over 1M	[34]
3	nuScenes [218]	Vehicles, pedestrians, cyclists, traffic signs, lights, road markings, and more	Over 1.4M	[35,142,146,147,150,153,163,165]
4	GRAM [38]	Vehicles, pedestrians, cyclists	Around 1M	[38]
5	GRAM-RTM [36]	Vehicles, pedestrians, cyclists, traffic signs, lights, road markings, and more	-	[36]
6	UA-DETRAC [36,219,220]	Car, bus, van, and others	8550	[37]
7	CDNet [221]	Cars, pedestrians, animals, buildings, trees, traffic signs, background scenes, and more	93,702	[38]
8	VEDAI [222]	Car, bus, truck, motorcycle, bicycle, pedestrian, traffic light, signs, buildings, vegetation, background	33,360	[44]
9	DAWN [223]	Person, car, bus, truck, motorcycle, bicycle, pedestrian, traffic light, signs, trailer, pole, buildings, vegetation, sky, ground, and unknown	275,350	[46,54]
10	MS-COCO [224]	Car, person, bicycle, motorcycle, bus, truck, train, stop sign, fire hydrant, traffic light	Over 2M	[46,55,105]
11	OSM [225]	No fixed categories	-	[49]
12	DroneVehicle [226]	Car, truck, bus, van, freight car	24,358	[51]
13	Highway Dataset [227]	Vehicles, pedestrians, bicycles, traffic signs, construction, and other objects	42,000	[33,55]
14	Space Cup Competition [228]			[228]
15	CityPersons pedestrian detection benchmark [229]	Pedestrians	3475	[60,70]
16	PETS2009 [230]	People, bicycles, motorcycles, cars, vans, trucks, and other vehicles	4005	[71]
17	CalTech Lanes Dataset [231]	People, bicycles, motorcycles, cars, vans, airplanes, faces, Frisbee, trucks, and more	30,607	[72,131]
18	Multispectral pedestrian detection [232]	Pedestrians	86,152	[73,74,75,76,79]
19	Aerial Infrared Pedestrian Detection Benchmark [80]	Pedestrians	Over 100K	[80]
20	GTSRB [233]	Traffic signs	51,839	[82,83,84,85,86,87,88,89,93,98]
21	BTSC [234]	Traffic signs	3740	[93]
22	LISA [235]	Traffic signs	6160	[97,169]
23	ITSRB & ITSDB [98]	Traffic signs	500	[98]
24	Cure-TSD [236]	Traffic signs	1080	[100]
25	Tsinghua-Tencent 100K [237]	Traffic signs	100,000	[102]
26	CCTSDB [238]	Traffic signs	7717	[104]
27	HRRSD [239]	Traffic signs	58,290	[104]
28	CuLane [240]	Lane marking, traffic signs, dazzle lights, and more	10,2448	[116,117,119,122,124,128,132,134,135,137]
29	TUSimple [241]	Vehicles, lane markings, traffic signs, pedestrians, cyclists, and more	12,224	[116,119,122,123,124,125,126,130,132,133,137,138]
30	BDD100K [242]	Pedestrians, riders, cars, trucks, buses, traffic signs, and more	1,407,782	[116]
31	Udacity Machine Learning Nanodegree Project Dataset [243]	Vehicles, lane markings, traffic signs, pedestrians, cyclists, and more	242,999	[139,144]
32	LLAMAS Dataset [244]	Car, bus, truck, motorcycle, bicycle, pedestrian, traffic lights and signs, yield light, and more	1300	[122]
33	Cracks and Potholes in Road Images Dataset [245]	Cracks and potholes	3235	[139]
34	Waymo Open Dataset [246]	Vehicles, pedestrians, cyclists, and signs	5,447,059	[148]
35	ETH Pedestrian Dataset [247]	Pedestrians, cyclists, cars, and van	61,764	[170]

## Data Availability

No data used in the article but only the state-of-the-art publications as listed in the ‘References’ section.
